# Comprehensive approach integrating water quality index and toxic element analysis for environmental and health risk assessment enhanced by simulation techniques

**DOI:** 10.1007/s10653-024-02182-1

**Published:** 2024-08-31

**Authors:** Mohamed Hamdy Eid, Mahmoud Awad, Essam A. Mohamed, Tamer Nassar, Mostafa R. Abukhadra, Ahmed M. El-Sherbeeny, Attila Kovács, Péter Szűcs

**Affiliations:** 1 Institute of Environmental Management, Faculty of Earth Science, University of Miskolc, 3515 Miskolc- Egyetemváros, Hungary; 2 Geology Department, Faculty of Science, Beni-Suef University, Beni-Suef, 65211 Egypt; 3 Faculty of Earth Science, Beni-Suef University, Beni-Suef, 62511 Egypt; 4 Geology Department, Faculty of Science, Cairo University, Cairo, Egypt; 5 Industrial Engineering Department, College of Engineering, King Saud University, P.O. Box 800, 11421 Riyadh, Saudi Arabia

**Keywords:** PTEs, WQI, Environmental and health risk, Monte Carlo simulation, Beni-Suef quaternary aquifer

## Abstract

Due to water shortages and the potential impact of Ethiopia’s new dam on the Nile River, Egypt is seeking new water resources. This study assesses the drinking water quality and associated risks from potentially toxic elements (PTEs) in the Quaternary aquifer (QA) in Beni-Suef, Egypt. Using a comprehensive approach, including PHREEQC geochemical modeling, ionic ratios, multivariate statistical analyses, and the integrated weight water quality index (WQI), the study evaluated the sources of ion contamination and the mixing of Nile water with QA. Various indices, such as the Heavy Metal Pollution Index (HPI), ecological Risk Index (RI), Hazard Quotient (HQ), and Hazard Index (HI), were used to assess ecological and health risks. Monte Carlo simulations provided probabilistic assessments of non-carcinogenic risks for adults and children. GIS tools were used to map risk indices, identifying the most deteriorated locations for sustainable management. The hydrochemical analysis revealed water facies including Na–Cl, Ca–Mg–HCO_3_, and mixed types, influenced by carbonate dissolution, ion exchange, and silicate weathering. Contamination sources, particularly in the north and south, were linked to agricultural activities, irrigation return flow, municipal waste, and evaporation. The WQI indicated that 10.14% of samples were extremely poor, 21.7% were poor, 26% were medium, and 42% were good to excellent. PTE contamination varied, with HPI values indicating good water quality in the central area in 53.6% of the collected samples (HPI < 30), but contamination in the north and south is high (HPI > 51). Ecological Risk Index values were below the threshold in 100% of samples (RI < 30), confirming water safety regarding PTEs. In comparison, for hazard index (HI) through oral/ingestion, adults exhibited HI values ranging from 0.012 to 2.16, while children showed higher values, ranging from 0.045 to 8.25. However, the hazard index for oral/ingestion exceeded safe limits in the north and south (HI oral > 1), posing non-carcinogenic risks. Monte Carlo simulations revealed significant risks from oral exposure to manganese (HQ oral > 1), particularly in El-Wasta and El-Fashn, necessitating further treatment and management.

**Supplementary Information:**

The online version contains supplementary material available at 10.1007/s10653-024-02182-1.

## Introduction

In developing countries, water shortage, climate change, quick industrialization, economic expansion, and urbanization are primary drivers of growing environmental pollution, creating national and international concerns (Abukhadra et al., 2022; Ata et al., 2024; Bin Jumah et al., 2021; Salam et al., 2021; Szűcs et al., 2024). In recent decades, the demand for water has significantly risen due to population growth, increased household consumption, and the expansion of industrial and agricultural activities (Egbueri et al., 2024; Karmakar et al., 2023; Khan et al., 2023). Industries such as petroleum-based chemicals and heavy automotive production produce contaminants such as heavy metals, organics, microplastics, pesticides, and newly developed pollutants, which endanger human health, groundwater resources, sustainable development, and environmental organizations (Bellucci et al., 2022; Saeed et al., 2023a, 2023b). Recent global investigations have discovered groundwater pollution from metals such as manganese, iron, copper, and zinc (Eid et al., 2024a, 2024b; Gad et al., 2023; Saeed et al., 2023b). Because of the enormous effects on individual issues, the global fear over pollutants in the atmosphere has grown. These hazardous metals are infamous poisons capable of causing organ damage and demonstrating teratogenic and carcinogenic tendencies(Githaiga et al., 2021; A. Mohammadpour et al., 2023a, 2023b; Amin Mohammadpour et al., 2023a, 2023b).

Heavy metal pollution in groundwater has been linked to major health difficulties such as degenerative neurological illnesses, renal damage, cardiovascular and respiratory ailments, and cancer (Abu et al., 2024; Eid, et al., 2024a, 2024b; Gad et al., 2023; Yassin et al., 2024). Potential toxic elements (PTEs) are inherently persistent and accumulate in groundwater, making it a major source of exposure for humans. As a result, public agencies frequently check PTE concentrations to reduce any health risks (Eid et al., 2024a, 2024b). Given the crucial importance of groundwater and the issues it faces, this study explores several forms of dangers to individuals and the environment posed by heavy metal contamination in the Beni-Suef region. Although copper (Cu), zinc (Zn), iron (Fe), and manganese (Mn) are required for metabolic activities, their concentrations in drinking water exceed permissible limits, posing health risks. Heavy metals can enter the body through eating, cutaneous contact, or inhalation (Eid et al., 2024a, 2024b; Gad et al., 2023; Saeed et al., 2023b). These contaminants are found in potable water sources such as groundwater and surface water, vegetables, and the air (Ata et al., 2024; Jafarzadeh et al., 2022; Kiani et al., 2021). Industrial operations, medicinal, residential, agricultural, and technological events are the primary causes of these metals’ presence in the environment. When heavy metal concentrations in drinkable water exceed the criteria specified by global organizations, it can cause severe health risks (Kazemi Moghaddam et al., 2022; Marufi et al., 2022). Comprehensive examinations of water quality are required to protect both the environment and human health. This begins with assessing water quality and identifying pollution sources in order to reduce contamination.

Proven approaches for measuring the ecological, environmental, and individual health risks associated with PTEs (Fe, Cu, Mn, and Zn) include many indices (MI, RI, HPI, HQ, and HI) (Egbueri et al., 2024), all of which can be improved by incorporating Monte Carlo simulations for increased accuracy and dependability (Githaiga et al., 2021; Mohammadpour et al., 2023a, 2023b). Furthermore, principal component (PC) analysis, as well as cluster analysis, are important approaches for scaling heavy metal pathways and understanding hydrochemical processes in groundwater and surface water (Abu et al., 2024; Eid et al., 2023, 2024a, 2024b; Flores et al., 2023; Gaagai et al., 2023; Salem et al., 2023; Szabó et al., 2023; Szűcs et al., 2024). Globally, groundwater supplies are severely contaminated and depleted, a problem that particularly affects the Quaternary flood plain, where Beni-Suef relies heavily on groundwater for consumption and agriculture (Awad et al., 2022). According to reports, more than one billion people in developing countries lack access to safe drinking and agricultural water, resulting in approximately 25,000 deaths per year (Onyebuchi Okafor et al., 2024).

Determining the sustainability of groundwater, a crucial water supply in arid regions, is complex due to problems like overuse, decreasing water levels, and the deterioration of water quality (Abu et al., 2024; Boualem & Egbueri, 2024; Egbueri et al., 2024; Karmakar et al., 2023; Khan et al., 2023; Yassin et al., 2024). A variety of methodologies have been used for determining groundwater quality, among which are the fuzzy comprehensive evaluation method (Zhang et al., 2020; Zhong et al., 2022) and the matter-element extension method (Wang et al., 2019). The entropy-based weighted technique is used for obtaining the weights of hydrochemical variables in groundwater, which aids in water quality examinations. This method reduces the number of components examined, accurately defines water quality types, and determines if the measured variables meet decision-making requirements for certain functional areas (Ikram et al., 2024; Islam et al., 2017). The entropy-weighted water quality index (EWQI) accurately evaluates the purity of groundwater, enabling ranking based on groundwater quality parameters (Amiri et al., 2014; Islam et al., 2017).

Egypt is currently experiencing water scarcity due to an increasing population and the predicted reduction in Nile River flow share due to the buildup of the Grand Ethiopian Renaissance Dam (GERD) (Elsayed et al., 2020). To satisfy expanding demand and manage the water scarcity both surface and groundwater have been heavily exploited, resulting in water supply challenges. A systematic hydrogeochemical and multivariate statistical analysis was carried out to better understand the factors that influence groundwater chemistry and pollutant sources in the western part of Beni-Suef region. Employing statistical methods in conjunction with geochemical tools aids in identifying the different mechanisms influencing groundwater ionic compositions, as well as hydrogeochemical change and origins of contamination (Al-Mashreki et al., 2023; Eid et al., 2024a, 2024b; Flores et al., 2023; Ibrahim et al., 2023). This combination technique is useful in identifying potential ionic sources, which aids in effective water quality management.

This study aims to comprehensively investigate the ecological and individual health issues associated with potentially toxic elements (PTEs) in the groundwater of the Beni-Suef Quaternary aquifer (QA). The research objectives are as follows: (1) To identify potential pollution sources using various statistical approaches, including principal component analysis (PCA), cluster analysis, ionic ratio analysis, and inverse distance weighting (IDW) interpolation. (2) To elucidate the geochemical processes governing water chemistry in the investigated area. (3) Application of integrated weight water quality index (WQI) method to detect suitability of different water resources for drinking. (4) To employ a novel technique combining various water quality criteria and indicators (WQI, HPI, HQ, RI, and HI) with deterministic models or probability-based techniques, notably the Monte Carlo approach, to assess non-carcinogenic health risks from PTEs contamination. The integration of these methodologies represents a significant advancement in the assessment of PTE contamination within the Beni-Suef Quaternary aquifer (QA). Although several studies were performed in the study area to investigate only the hydrochemical evaluation (Awad et al., 2022), the current study is the first investigation fill the gap of the health risk regarding toxic elements with simulation technique to decrease the uncertainty and applying advanced approach integrate between hydrochemistry, ion source detection, water quality using integrated weight water quality index.

## Materials and methods

### Study area and geographic description

**Fig. 1:**
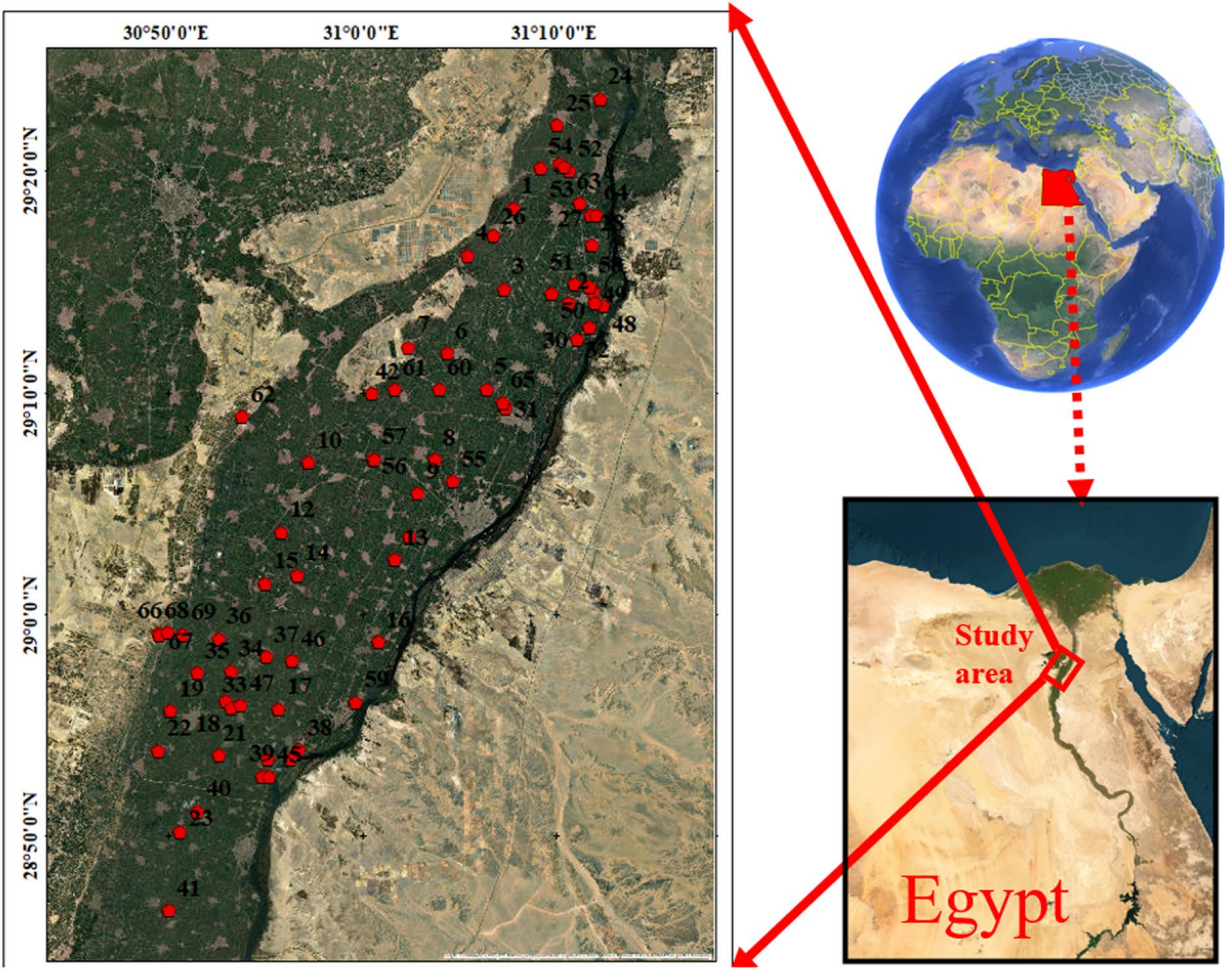
Location map of Beni-Suef area

Beni-Suef Governorate within the Nile Valley occupies an area of approximately 10,950 km^2^ bounded by the Eastern and Western Deserts (Melegy et al., 2014). About 12% of the area is inhabited, while the remaining percentage 88% is desert lands. Agricultural lands represent 85% of the total populated area. Beni-Suef Governorate is located between latitudes 28° 45′ and 29° 25′ N and longitudes 30° 49′ and 31° 18′ E (Fig. 1). It includes seven towns namely, Beni-Suef (the capital), El-Wasta, Naser, Beba, Ihnasia, Somosta, and El-Fashn. Northwards, it is bordered by the Giza Governorate and southward by El-Minia Governorate. It lies between El-Fayoum Governorate to the west and the Red Sea Governorate to the east (Awad et al., 2022).

### Aquifer system

**Fig. 2:**
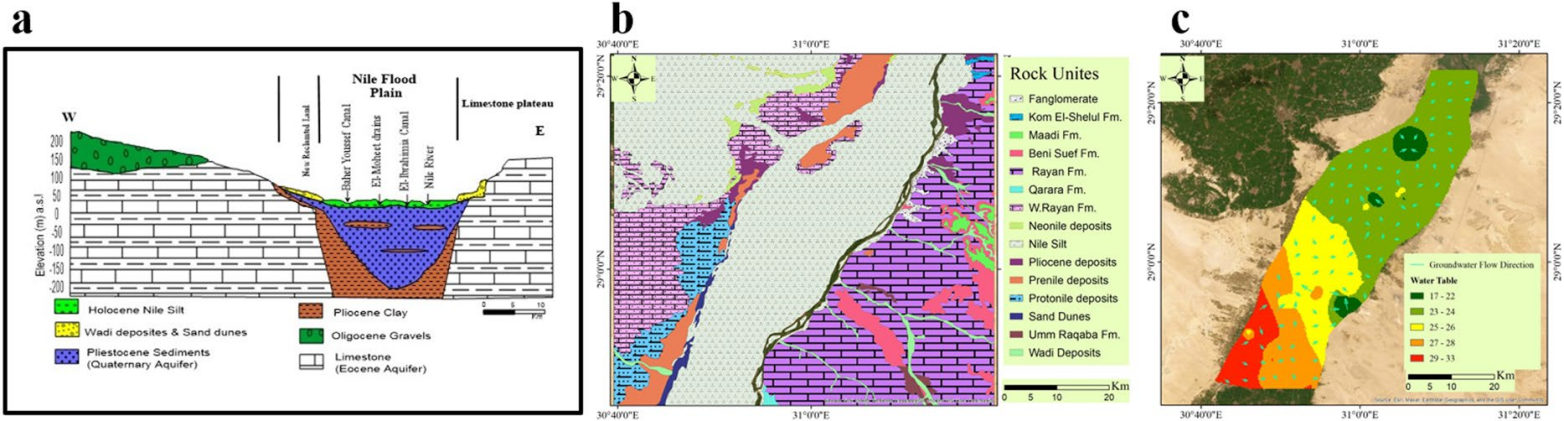
Hydrogeological cross-section of Quaternary and Eocene aquifers (Awad et al., 2022) (**a**), surface geology (**b**), and ground water flow direction (**c**) at Beni-Suef area

Within the Nile Valley region, regional and local aquifers are encountered (Awad et al., 2022; Melegy et al., 2014; Said, 2013, 2017) which are summarized as follows:

1. Quaternary aquifer system. It consists of Pleistocene graded sands and gravels; it is covered with Holocene silt and clay (semi-confining) with a thickness varying between 0 and 20 m (Fig. 2a). In the desert fringes, outside the floodplain, the semi confining layer vanishes, and phreatic conditions prevail. The aquifer is underlain by Pliocene clay. The aquifer thickness ranges from 0 (near the fringes), to more than 250 m in the centre (Sohag and El-Minia). The saturated thickness of the aquifer ranges from 0 to more than 200 m. The aquifer transmissivity ranges from 20,000 m^2^/day in the centre of the floodplain, to less than 500 m^2^/day at the edges.
2. Nubian Sandstone aquifer is the second regional aquifer, which extend from Qena southward, being confined by clays and shales in the north. The body of the aquifer (Cretaceous rocks) slopes from ground level (south of Aswan) to 2,000 m below mean sea level near Cairo. The saturated thickness ranges from a few meters to more than 1,000 m. The Transmissivity ranges from 500  to 6,000 m^3^/day.
3. Local aquifers constitute Quaternary and Tertiary deposits and represent a moderate source of groundwater in the wadis and along the fringes of the Nile Valley. They are recharged from the occasional rainfall storms, and receive recharge by upward leakage from the underlying deep aquifers. They are generally phreatic, semi confined to confined. Their thicknesses vary from one place to another, ranging from few meters close to the limestone plateau to more than 200 m near the Nile Valley. The surface geology of the study area is illustrated in Fig. 2b showing the different geological composition and formations. The local ground water flow in the study from south west to north east with cone of depression in the water table exhibited in the north and central part of Beni-Suef area (Fig. 2c).

### Samples collection and analytical approaches

In 2020, an overall of 69 water samples were methodically gathered from groundwater production well’s locations (Fig. 1) To guarantee uniformity, samples were collected after 10 min of production, ensuring comprehensive representation from each investigated point. Before sampling, 500-ml polyethylene containers were extensively cleansed using chemicals, completely rinsed with filtered water, and submerged in a 10% HNO_3_ solution overnight. The obtained samples were then taken to a laboratory with a regulated temperature of 4 °C for further analysis. On-site pH measurements, temperature (°C), (TDS), (EC), were conducted using specialized instruments: pH meter, and a digital thermometer (Hannah, Woonsocket, RI, USA). Additionally, TDS and EC were analyzed utilizing digital TDS and EC meters (HM digital, Redondo Beach, CA, USA). To ensure accuracy, all digital meters underwent standardization with deionized water and buffer solutions before the commencement of sample analysis. For cations analysis, the samples underwent filtration through 0.45 µm filters. Subsequently, 10 drops of ultra-pure HNO_3_ were added to one set of samples. Calcium (Ca^2+^) and magnesium (Mg^2+^) contents were assessed using the EDTA titrimetric method, which employs ethylenediaminetetraacetic acid. Sodium (Na^+^) and potassium (K^+^) ion contents were measured utilizing a flame photometer (ELEX 6361, Eppendorf AG, Hamburg, Germany). Total hardness (TH) was evaluated using Eriochrome Black-T (C20H12N3O7SNa) and ammonium chloride (NH_4_Cl) indicators in an EDTA solution. To assess chloride (Cl^−^) concentrations, a titration method employing silver nitrate (AgNO_3_) and potassium chromate (K_2_CrO_4_) indicators was employed. For the detection of bicarbonate (HCO_3_^−^) and carbonate (CO_3_^2−^) concentrations, a titrimetric technique involving the solution of sulfuric acid (H_2_SO_4_) and methyl orange indicator was utilized. Additionally, Cl^−^ concentrations were determined through titration with silver nitrate. Concentrations of sulfate (SO_4_^2−^) and nitrate (NO_3_^−^) were tested using a spectrophotometer based on the visible ultraviolet (UV) spectrum (DR/2040-Loveland, CO, USA). Fe, Cu, Zn and Mn were assessed through flame atomic absorption spectrometry (FAAS).

#### Quality assurance and control

The water quality analysis followed the standard methodology specified by the American Public Health Association (APHA) in 2012. To ensure the accuracy of on-site testing equipment, we carefully standardized all instruments with deionized water and buffer solutions before starting sample analysis. Various quality assurance procedures were applied during the water sample examination. The analytical processes were validated by instrument calibration, accuracy, and predictability evaluations. Charging balance errors (CBE) were evaluated following field observations and then validated in the laboratory. The samples were examined in triplicate, and the average values were given as well. Equation (1) was used to analyze anion–cation balance errors based on the neutrality principal, which states that the sum of the number of cations equals the sum of the number of anions in meq/L. The CBE for all examined samples was within the permissible range of ± 5%.

1 $${\text{CBE}} = \frac{{\Sigma {\text{Cations}} - \Sigma {\text{Anions}}}}{{\Sigma {\text{Cations}} + \Sigma {\text{Anions}}}}*100$$

Furthermore, the quality assurance of the analytical procedure was double-checked through a meticulous examination involving Certified Reference Material (CRM) and the blank technique analysis (Tables S1, S2).

The different metal contents in the sample solutions were obtained using a curve for calibration. To calibrate the device, a 50-ml (mL) intermediate standard was employed as an operational standard for Four toxic metals. Table S3 shows the concentrations of intermediate, operating standards, and coefficient of correlation values for each metal. When the correlation coefficient could be more than 0.999, it indicated that the relationship was strong. The measured amount of every metal in the collected sample was determined using interpolation of calibration curves. Every examination was conducted in triplicate.

The abundance of toxic metals in each sample was examined using FAAS. The lamp’s current, slit width, and wavelength of the apparatus have been adjusted to deliver the lowest possible intensity of signal, as described in Table S4. The principal line sources were hollow cathode lamps operated in accordance with the manufacturer’s specifications. Acetylene along with airflow rates were provided to ensure that the instrument had the proper flame settings. The metallic elements were identified using absorption/concentration manner, and the results were manually recorded. The same approach was used to analyze the metal found in the spiked sample.

The methods employed were validated by performing limit of detection and quantification (LOD and LOQ), accuracy, precision, and recovery testing based on the following equations below;

$${\text{LOD}} = \, \left( {{3}*{\text{standard deviation of blank method}}} \right)$$

$${\text{LOQ}} = \, \left( {{1}0*{\text{standard deviation of blank method}}} \right)$$

The variance and % of relative standard deviation (RSD) were used to determine the results’ accuracy and precision. The RSD of triplicate readings for every single sample was applied to calculate precision as described in the equation below;

$$\% {\text{RSD}} = \, \left( {{\text{SD}}/{\text{mean}}} \right) \, *{1}00$$

### Hydrochemical evaluation method

In this investigation, the chloralkaline indices (CAI-I and CAI-II) (Eqs. (2–3) were employed to ascertain the mineral composition within the aquifers and the ionic exchanges occurring in groundwater systems.

2 $$CAI-I=\frac{{Cl}^{-}-({Na}^{+}-{Ca}^{+})}{{Cl}^{-}}$$

3 $$CAI-II=\frac{{Cl}^{-}-({Na}^{+}-{Ca}^{+})}{{So}_{4}^{2-}+{HCO}_{3}^{-}+{CO}_{3}^{2-}+{NO}_{3}^{2-}}$$

The hydrochemical evaluation methods included, Gibbs (Gibbs, 1970), Piper (Piper, 1944), CAI-I, CAI-II (Schoeller, 1977), and Ionic ratios (Tlili-Zrelli et al., 2013)plots to determine the water type of different water resources and the mechanism controlling the water chemistry. The graphs were visualized using Digramme software and an Excel file. Geochemical modeling based on physicochemical characteristics and heavy metals was used to assess the mineral saturation status and which minerals contribute the most to the enrichment of elements in water via water–rock interactions. The model extracted from PHREEQC produced a saturation index (SI) for each mineral (Parkhurst & Appelo, 1999; Plummer et al., 1988), with a positive value indicating super saturation and a negative value indicating under saturation, while a zero value indicates an equilibrium state in which water is unable to dissolve or precipitate certain minerals. The saturation index was determined based on standard equations 57,58 utilizing log ion activity product divided by solubility product (Eq. 4).

4 $$({\text{SI }} = {\text{ log }}\left( {{\text{IAP}}/{\text{K}}} \right)$$

IAP stands for “ion activity product,” and ${K}_{sp}$ stands for “solubility product” at a given temperature.

### Drinking water quality index (WQI) using integrated weight

The Integrated Weight Water Quality Index (WQI) is a ranking system that uses the Water Quality Index to assess the total impact of physicochemical variables on water quality. In this study, the WQI was used to evaluate water quality (Al-Asad et al., 2023; Gao et al., 2020). The WQI is calculated in five steps: entropy weighting, CRITIC-based weighting, determining integrated weights, computing the integrated-weight water quality index, and evaluating groundwater quality using WQI values.

#### Entropy weight calculation

The entropy-weighted water quality index (EWQI) is a method used to estimate water quality by calculating an overall entropy value (Adimalla, 2021; Shannon, 1948) based on specific hydrochemical variables. The EWQI calculation process consists of three phases, which are detailed below:

**In Step 1**, We computed the eigenvalues of the matrix X using Eq. (5), where m and n denote the total number of investigated samples and hydrochemical parameters to be evaluated, respectively.

5 $$\text{x}=\left[\begin{array}{ccc}{\text{x}}_{11}& {\text{x}}_{12}& {\text{x}}_{1\text{n}}\\ {\text{x}}_{21}& {\text{x}}_{22}& {\text{x}}_{2\text{n}}\\ {\text{x}}_{{\text{m}}_{1}}& {\text{x}}_{{\text{m}}_{2}}& {\text{x}}_{\text{mn}}\end{array}\right]$$

**In Step 2**, We rely on Eqs. (6) and (7) for determining the standard matrix Y. As hydrochemical indicators have considerable dimensional discrepancies, data normalization is essential before calculating the EWQI. Here, (*Xij*)*x* denotes the maximum value, while (*Xij*)*min* represents the smallest value for the associated hydrochemical parameters.

6 $$Yij=\frac{Xij-\left(Xij\right)min}{(Xij)max-\left(Xij\right)min}$$

7 $$\text{Y}=\left[\begin{array}{ccc}{\text{Y}}_{11}& {\text{Y}}_{12}& {\text{Y}}_{1\text{n}}\\ {\text{Y}}_{21}& {\text{Y}}_{22}& {\text{Y}}_{2\text{n}}\\ {\text{Y}}_{{\text{m}}_{1}}& {\text{Y}}_{{\text{m}}_{2}}& {\text{Y}}_{\text{mn}}\end{array}\right]$$

**In Step 3**, We leverage Eqs. (8–10) to compute information entropy (ej) and entropy weight (wj). Pij reflects sample I index j value.

8 $$ej=-\frac{1}{Lnm}{\sum }_{i=1}^{m}\left(\text{Pij}\times \text{LnPij}\right)$$

9 $$Pij=\frac{Yij}{{\sum }_{i}^{m}\left(\text{Yj}\right)}$$

10 $$Wj1=\frac{1-ej}{{\sum }_{i=1}^{n}\left(1-ej\right)}$$

#### Objective weight (CRITIC method)

In this study, the criteria’s importance The Inter-criteria Correlation (CRITIC) technique was used to determine the objective weights of variables and overcome the limitations of traditional information entropy methods. The objective weight can be calculated using the following equation (Eqs. 11–13):

11 $${r}_{ij}=\frac{\sum \left({x}_{ij}-\overline{{x }_{ij}}\right)\left({y}_{ij}-\overline{{y }_{ij}}\right)}{\sqrt{\sum {\left({x}_{ij}-\overline{{x }_{ij}}\right)}^{2}\Sigma {\left({y}_{ij}-\overline{{y }_{ij}}\right)}^{2}}}$$

12 $$Sj=\updelta j{\sum }_{i=1}^{m}\left(1-\text{rij}\right)$$

13 $$Wj2=\frac{Sj}{{\sum }_{j=1}^{m}\left(Sj\right)}$$

In this context, wj2 represents the objective weight of the jth parameter, with m denoting the total number of variables. Sj represents the quantity of information, while δj represents the standard deviation of the jth parameter.

#### Integrated-weight estimation

The following equation (Eqs. 14–16)is utilized to determine the integrated-weight Wj:

14 $${\text{W}}_{j} \, = \,pwj{1}\, + \,({1}\, - \,p)wj{2}$$

15 $$p={\sum }_{j=1}^{m}\left[{\left({W}_{j}-{w}_{j1}\right)}^{2}{\left({W}_{j}-{w}_{j}2\right)}^{2}\right]$$

16 $$Wj=\frac{wj1\times wj2}{{\sum }_{j=1}^{m}\left(wj1\times wj2\right)}$$

In this section, p represents a preference coefficient, with pϵ [0,1].

#### Drinking water quality index (WQI) using integrated Weight

After estimating the entropy weight, wj1, and the objective weight, wj2, the following formulas (Eqs. 17, 18) are used for calculating the Integrated Weight Water Quality Index (WQI):

17 $$Qj = \frac{Cj - Cjp}{{Sj - Cjp}} \times 100$$

18 $$DWQI={\sum }_{j=1}^{m}\left(WjQj\right)$$

In the equations, j represents the experimental concentration of each parameter in mg per liter, while Cjp is the variable’s standard value in pure drinking water. All variables are zero, with the exception of pH, which has a standard score of seven. The standard value (Sj) for each physicochemical factor evaluated using WHO standards (WHO 2017) is presented in mg/L. Table 1 shows the input parameters and the Integrated Weight.

**Table 1:** The parameters used in calculation of integrated weight water quality index (WQI)

Parameter	Unit	Cjp (variable’s standard value in pure water)	Sj (WHO 2017)	Wj (integrated weight)
TDS	mg/L	0	1000	0.042538
pH		7	7.5	0.085516
EC	µS/cm	0	1500	0.038892
Na^+^	mg/L	0	400	0.038133
K^+^	mg/L	0	12	0.090798
Mg^2+^	mg/L	0	150	0.04749
Ca^2+^	mg/L	0	200	0.045792
Mn	mg/L	0	0.1	0.118172
Fe	mg/L	0	0.3	0.154696
Cl^−^	mg/L	0	600	0.048279
SO_4_^2−^	mg/L	0	400	0.056017
HCO_3_^−^	mg/L	0	200	0.100238
NO_3_^−^	mg/L	0	45	0.059186
TH	mg/L	0	500	0.074253
				∑Wi = 1

Water is divided into five categories based on WQI values (Al-Asad et al., 2023; Gao et al., 2020). When the WQI result falls below 100 (excellent to good), the water is considered safe for oral consumption and other applications. Medium or intermediate quality ranges from 100 to 150, while bad and extremely poor water quality ranges from 150 to more than 200.

### Heavy metal pollution index (HPI)

The Pollution Index (HPI) is a valuable metric for assessing the level of heavy metal pollution in water bodies (Al-Hejuje et al., 2017). It is particularly effective in determining the suitability of water for consumption in the presence of heavy metals. The HPI is calculated using attribute ratings and weighted mean calculations. Each pollutant characteristic is assigned a weight, and a grading system ranging from 0 to 1 underscores the significance of each quality aspect or its alignment with specified reference standards. The specific computations for determining the HPI are provided in Eqs. 19 and 20 (Shankar, 2019).

19 $${\text{HPI}} = \frac{{\sum _{{{\text{i}} = 1}}^{{\text{n}}} {\text{W}}_{{\text{i}}} {\text{Q}}_{{\text{i}}} }}{{\sum\nolimits_{{{\text{i}} = 1}}^{{\text{n}}} {{\text{Wi}}} }}$$

In the formula, Qi is the sub-index factor, n represents the number of analyzed variables, wi is the weight assigned to each factor, calculated as 1/Si, where Si is the standard value for each variable. Qi is also the sub-index of the boundary, as defined by Eq. 17.

20 $${Q}_{i}={\sum }_{i=1}^{n}100 \times \frac{{C}_{i}}{{S}_{i}}$$

The HPI indicator determines the amounts of the elements iron (Fe) and manganese (Mn). The metals index is frequently examined using a modified five-category scale. Water quality is categorized as excellent (HPI < 15), good to intermediate (15 < HPI < 30), poor to unsuitable (HPI > 30), very poor (76 < HPI < 100), and unsuitable (HPI > 100) (Edet & Offiong, 2002; Qu et al., 2018).

### Ecological risk index

The ecological risk indicator (RI) for hazardous metals, first developed by Hakanson, is a tool for determining the risk associated with an excess of heavy metals in an ecosystem. As highlighted by Xie, this indicator takes into account heavy metal concentrations, kinds, sensitivity, toxicity, and background levels. While useful to a variety of scientific domains, it was specifically used in this study to assess the ecological risks associated with heavy metals in groundwater. The formula for computing the RI is presented below (Eq. 21).

21 $$RI=\sum {E}_{r}^{i}={T}_{r}^{i}\times \left\{\frac{{c}^{i}}{c{i}_{bg}}\right\}$$

In the equation, ${E}_{r}^{i}$ reflects a substance’s possible ecological indicator portion; ${T}_{r}^{i}$ depicts the specified metal’s toxic reaction variable (Table S5); ${c}^{i}$ designates a typical level of PTEs in each sample, and $c{i}_{bg}$ represents the background score or value of every metal (Table S5). The RI represents the whole ecological impact. The risk indicator (RI) is classified into four categories or levels of possible risk: low, moderate, significant, and very high, with RI values < 30, 30–60, 60–120, and > 120, respectively (Yuan et al., 2015).

### Multivariate statistical methods

Researchers frequently employ multivariate statistical methods to thoroughly understand groundwater condition and their fundamental chemistry (Eid et al., 2024a, 2024b; Gaagai et al., 2023; Saeed et al., 2023b). Our study combined trace metals, physical, and chemical attributes to investigate the intricate interactions among various factors and components within aquatic ecosystems. This framework used Principal Component Analysis (PCA) and Hierarchical Cluster Analysis (HCA) as analytical tools.

### Risk of exposure to potential toxic elements (PTEs)

Drinking water contaminated with toxic metals poses risks of both non-carcinogenic and carcinogenic ailments in humans (Bineshpour et al., 2021). This study followed procedures established by the U.S. Environmental Protection Agency (USEPA) to measure the non-carcinogenic hazards connected with Cu, Fe, Mn, and Zn (Selvam et al., 2022). The USEPA’s framework for health risk assessment, established in 2004, aims to evaluate the non-cancerous health risks posed by heavy metal factors in surface water and groundwater through ingestion, inhalation, and dermal contact. The primary hazard stems from directly ingested water and uptake via the skin (Saeed et al., 2023c). This approach estimates the effect of PTEs ingested by humans using the CDI method as described in Eqs. 22 and 23, respectively, (USEPA, 2004).

22 $${\text{CDI}}_{\text{oral}}=\frac{{\text{C}}_{\text{HMs}}\times \text{EF}\times \text{IR}}{\text{AT}\times \text{BW}} \times \text{ED}$$

23 $${\text{CDI}}_{\text{dermal}}=\frac{{\text{C}}_{\text{HMs}}\times \text{ET}\times \text{EF}\times \text{Kp}\times \text{SA}\times \text{CF}}{\text{BW}\times \text{AT}} \times \text{ED}$$

CDI refers to chronic daily intake (mg/kg/day), while *C*_HMs_ represent the concentration of each heavy metal (mg/L). The (IR) is the intake rate (children:1.8 L/day; adults: 2.2 L/day). The (ED) indicates the Exposure duration (children: 6 years; adults: 70 years) with an exposure frequency (EF) of 350 days per year for both adults and children. K_*p*_ denotes the permeability coefficient (cm/h), as indicated in Table S5; ET signifies exposure time (0.58 h/day for adults and 1 h/day for children). SA denotes the skin area (18,000 cm^2^ for adults and 6600 cm^2^ for children). CF represents the unit conversion factor (1 × 10^–3^ L/cm^3^). BW stands for body weight (children: 15 kg; adults: 70 kg). AT refers to the average duration of carcinogenic hazards (Saeed et al., 2023c; Selvam et al., 2022; Xu et al., 2020).

#### Non-carcinogenic risk assessment

This study evaluated the health hazards associated with Fe, Cr, Mn, and Pb (PTEs) in groundwater through risk assessment model created by USEPA. The non-carcinogenic risk index (HI) is a predictive model developed by the USEPA for assessing the health risks of chemical element combination. The HI comprises two components: chronic daily ingestion (CDI) and the hazard quotient (HQ), represented by the following equations (Eqs. 24–26):

24 $${HQ}_{dermal/oral}=\frac{{\text{CDI}}_{\text{dermal}}{/\text{CDI}}_{\text{oral}}}{{\text{RfD}}_{\text{dermal}}{/\text{RfD}}_{\text{oral}}}$$

25 $${RfD}_{dermal}={RfD}_{oral}\times ABS$$

26 $$HI={HQ}_{oral}+{HQ}_{dermal}$$

RfD (mg/kg/day) stands for the reference dose of a particular heavy metal. The RfD values for various heavy metals are provided in Table S5.

#### Monte Carlo simulation techniques

In this investigation, Monte Carlo was utilized as a simulation technique to estimate the probability distributions of various attributes such as PTEs levels, exposure time and frequency, ingestion rates, absorption coefficients, skin surface area, and individual body weight. This approach or method aims to characterize the probability distributions and uncertainty reduction (Eid et al., 2024a, 2024b; Qu et al., 2018; Saeed et al., 2023a, 2023b). This technique allows for the prediction of the hazard quotient (HQ) for both oral and dermal exposure in two age groups (children and adults). By integrating this simulation with the USEPA’s health risk assessment framework, we can assess the adverse effects of heavy metal exposure, estimating NCR probability distributions. The analysis includes variables such as heavy metal concentrations and other related factors, as detailed in Eqs. (22–25). To ensure the reliability of the simulation, 10,000 iterations were performed using Python. The consistency between actual and simulated HQ values calibrates the model’s efficiency. The distribution approach for PTEs levels was based on 2020 data, while variables like ingestion rate, exposure duration, skin surface area, and body weight were modeled using normal distributions to accurately represent their real-world distributions.

## Results and discussions

### Measured parameters statistics

The physicochemical properties of groundwater in the Beni-Suef Quaternary Aquifer (QA) are critical in the preliminary assessment of its quality and suitability for agricultural and potable use. This is an effective method for identifying environmental concerns, defining trends, and communicating insights into water resources, geochemical dynamics, and water quality. The following factors were used to determine the appropriateness of groundwater for drinking purposes within the selected shallow aquifer.

Meanwhile, the selected parameters like pH, EC, TDS, K^+^, Na^+^, Mg^2+^, Ca^2+^, Cl^−^, SO_4_^2˗^, HCO_3_^˗^, and NO_3_^˗^, and heavy metals (HMs) such as Fe, Zn, Cu, and Mn are altering the quality and productivity of the soil and could cause several environmental and health risks. The statistical methods of the analyzes parameters of 69 water samples were presented in Table 2.

**Table 2:** Statistical analysis of the different analyzed attributes in QA across the investigated area

Parameters	pH	EC (µs/cm)	TDS (mg/L)	TH (mg/L)	Cl^−^ (mg/L)	SO_4_^2−^ (mg/L)	Cu (mg/L)	Fe (mg/L)
Min	6.5	296	225	50	13	0.05	0.002	0.0002
Max	8.3	9850	4930	2288	2799	985	0.16	0.62
Mean	7.7	1755	982	345	258	141	0.049	0.152
STD	0.3	1458.4	800.7	397.2	432.3	156.9	0.038	0.160

Parameters	HCO_3_^−^ (mg/L)	NO_3_^−^ (mg/L)	Ca^2+^ (mg/L)	Mg^2+^ (mg/L)	Na^+^ (mg/L)	K^+^ (mg/L)	Mn (mg/L)	Zn (mg/L)
Min	65	0.001	2	8.06	14	1	0.002	0.003
Max	851	158	697	133	1600	74	1.71	0.88
Mean	387.5	7.3	98	35	209	15	0.422	0.081
STD	183.6	19.4	101.5	21.1	220.3	16.1	0.421	0.113

The above-mentioned parameters (Table 2) were compared with well-known drinking standard (WHO 2017) based on their concentrations in the groundwater of QA (Ben-Suef Quaternary aquifer) and using IDW interpolation method to determine the most deteriorated location from different sources of contaminations for future management and treatment scenarios. The simple statistics including minimum, maximum, mean, and standard deviation could give general differences and ranges of ions and metals concentration and refers to water quality situation.

Total dissolved solids (TDS), indicative of salinity, ranged from 225 to 4930 mg/L, with an average of 982 mg/L, surpassing the permissible drinking limit of 1000 mg/L in 26% of QA water samples. The distribution map of the TDS showed that the salinity of the ground water increase from east to west direction which indicate that there is possibility of mixing fresh water from the Nile river with brackish groundwater from QA decrease the salinity of water through dilution process in the eastern side of the study area. The western side of the study area has low contribution from the Nile river and high residence time of the water rock interaction could increase the groundwater salinity. The groundwater was classified as fresh water in 74% of samples and brackish in 26% of samples in QA. pH levels ranged from 6.5 to 8.3, indicating a neutral to slightly alkaline quality. Calcium concentrations fell within WHO drinking standards, ranging from 2 to 667 mg/L. it was noted that 93% of samples did not exceed the permissible limit (< 200 mg/L) for drinking, while 7% exceed the limits. Notably, five samples (S1, S60, S62, S67, and 69) located in western and northern part of investigated area exhibited the highest Ca^2+^ concentration. Magnesium (Mg^2+^) levels in all samples were generally within acceptable limits (< 150 mg/L) for drinking, showing no restriction regarding magnesium ion concentration. Sodium (Na^+^) concentrations ranged from 14 to 1600 mg/L, deemed safe for drinking in 96% of samples but unsuitable for drinking in 4% of samples (S1, S60, and 67), particularly in the south west and north west part of the study area. The predominant anions were sulfate (SO_4_^2−^) and chloride (Cl^−^), with concentrations ranging from 0.05 to 985 mg/L and 13 to 2799 mg/L, respectively. While these levels met drinking standards in 94% of samples for sulfates and 96% for chloride, they exceeded drinking limits in few samples located in south west and north west part of the study area. Bicarbonate (HCO_3_^−^) concentrations were acceptable for drinking in few samples (21.5%) and exceed the limits in 78.5% with value ranges between 65 and 851 mg/L.

Although nitrates availability in groundwater is linked to chemical pollution in the study area, their concentration was lower than 45 mg/L in most samples. It was noted that the maximum concentration of NO_3_^−^ exhibited in S67 with value 158 mg/L.

**Fig. 3:**
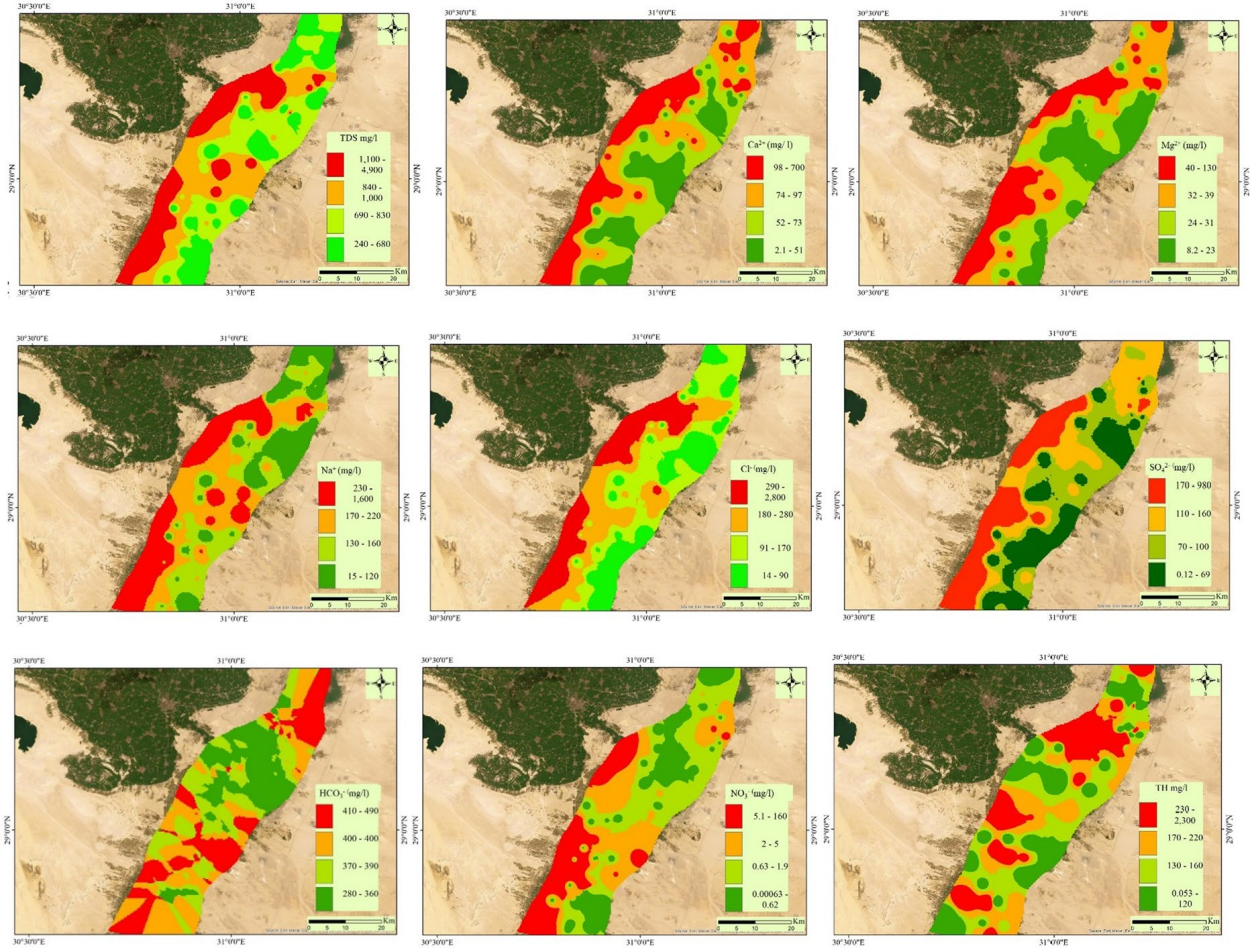
Distripution maps of the measured parameters in the groundwater samples of the Beni-Suef Quaternary aquifer (QA)

Potential toxic elements (PTEs) exhibited varied concentrations, with Fe, Mn, Zn, and Cu ranging from 0.0002 to 0.62 mg/L, 0.002 to 1.71 mg/L, 0.003 to 0.88 mg/L, and 0.002 to 0.16 mg/L, respectively. Concerningly, a significant proportion of samples exceeded the permissible drinking limits for Fe (16%), and Mn (56.5%), could cause negative environmental and health risks. On the other hand, Zn and Cu were within the safe limits of drinking in all samples. Distribution maps of measured parameters, depicted in Figure S1, highlight locations susceptible to water quality deterioration which was mainly in western part of the investigated region. The distripution maps of the measured parameters in the groundwater samples of the Beni-Suef Quaternary aquifer (QA) were ilustrated (Fig. 3) to show the spatial variation in the concentration of ions and physical parameters.

The distribution maps of measured parameters showed similar trend of increasing the concentration of TDS, Na^+^, Ca^2+^, Mg^2+^, TH, Cl^-^, and SO_4_^2−^ from east to west direction. This similar trend indicates that these parameters have similar water rock interaction condition and the dissolution of minerals such as calcite, dolomite, gypsum, halite, and anhydrite could increase the groundwater salinity, while the eastern side of study area is close to the Nile River and could receive recharge of fresh water make dilution. Incontrast the western side of the investigated area receive less fresh water from the Nile river and the groundwater rock interaction is the main mechanism governing the groundwater salinity. The rondum distribution of the NO_3_^−^ and HCO_3_^−^ in the study area compare to the other elements reveal that the anthropogenic activities increase the concentration of HCO_3_^−^ and NO_3_^−^ in the aquifer system.

### Hydrochemical characteristics

**Fig. 4:**
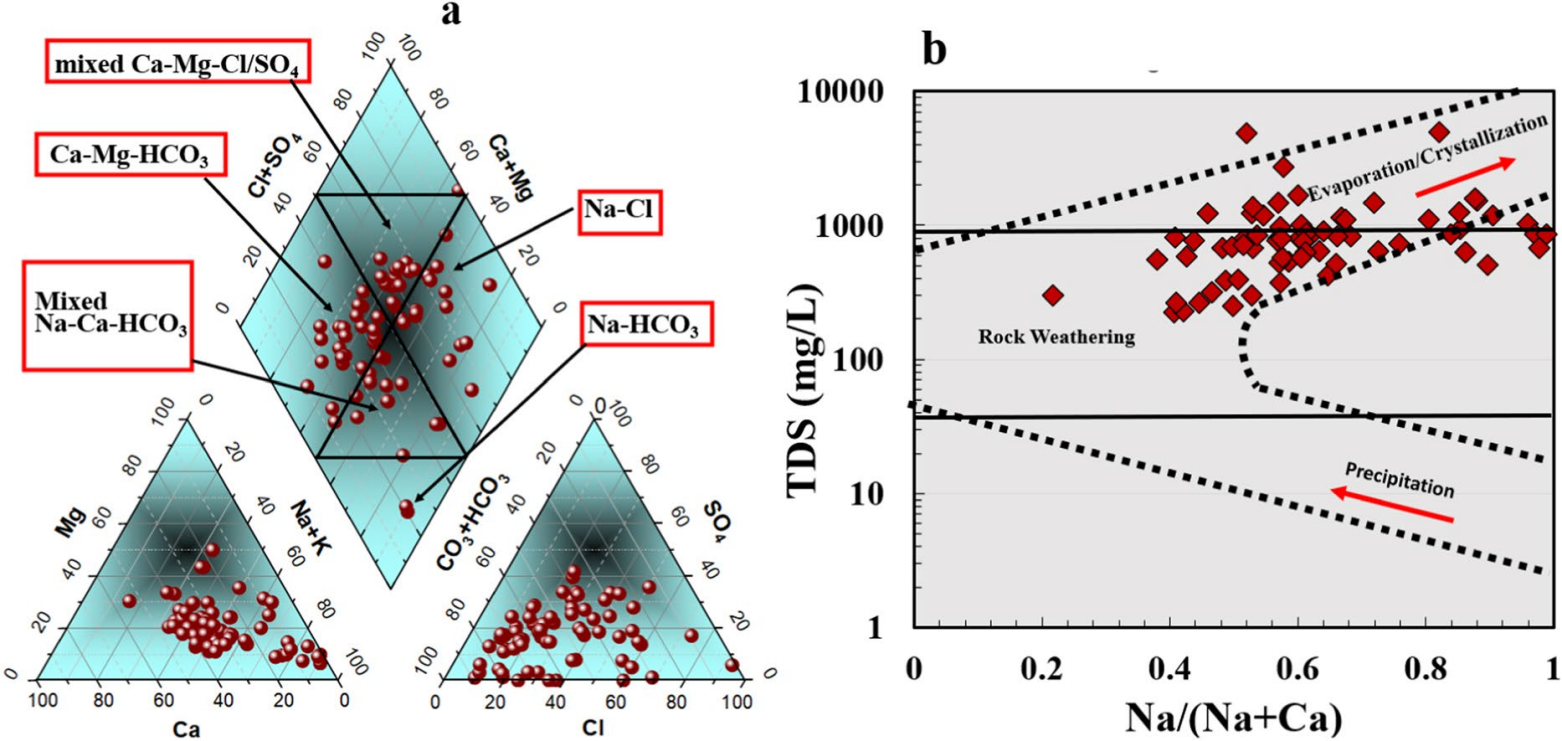
Piper plot **a** illustrates the water type/facies of all samples from different locations in Beni-Suef Quaternary aquifer, and Gibbs Plot showing the mechanisms controlling water chemistry (**b**)

A Piper plot, first developed by Piper, was used to identify the water types/facies found in water samples (Piper, 1944). The Piper scatter plot (Fig. 4a) divided the gathered samples into five separate facies. It was noted that 23.1% of samples representing the western part of investigated region fell in the Na–Cl facies zone, 34% fell in Ca–Mg–HCO_3_ category, 17.4% categorized as mixed Na–Ca–HCO_3_, 21.8% of samples fell into the mixed Ca–Mg–Cl/SO_4_ facies zone, and the rest 2.8% of samples represented by Na–HCO_3_. The distribution of samples within five different facies in piper plot reflect the wide range of water chemistry evolution in the Quaternary aquifer from the west to east and from south to north of the study area. These variations could be related to several factors including geological composition, anthropogenic activities, and mixing of different water resources specially the water samples closed to the Nile River. The mechanisms controlling water chemistry and contamination sources could be interpreted through multivariate statistical analyses with support of interpolation of various parameters in the following sections below where piper plot has limited information regarding water type only.

**Fig. 5:**
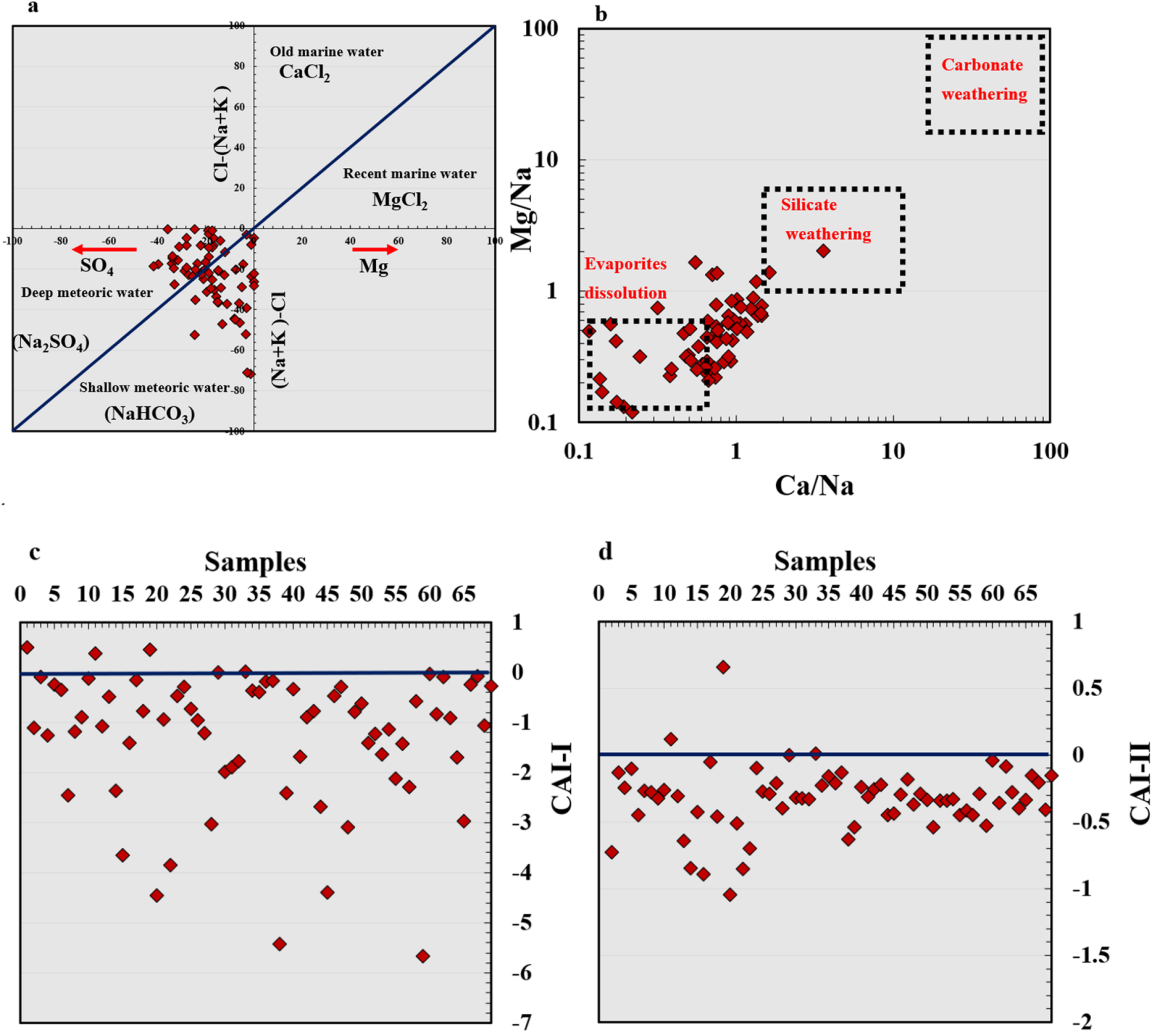
Sulin scatter plot and ionic ratios Mg^2+^/Na^+^ vs Ca^2+^/Na^+^ showing water origin mechanism control water chemistry in Quaternary aquifer (**a**, **b**), type of ion exchange using CAI (**c**, **d**)

The Gibbs chart or scatter plot (Gibbs, 1970) is an effective method for determining the impact of multiple processes on water chemistry, and it divides the diagram into three primary parts (Fig. 4b). The graphical representation shows that most samples are inside the zone of rock-weathering and the rest fell within evaporation/crystallization zone. The western part of study area is influenced more with evaporation/crystallization mechanism, while the central and eastern part in the direction of the Nile River the groundwater evolutes and the change in water type because of water–rock interaction, ion exchange and/or mixing with different sources. The Sulin graph (Fig. 5a) is a useful tool for determining groundwater origin and distinguishing between deep meteoric, shallow meteoric, old marine, and recent marine water based on the percentage of (Cl-(Na + K))/Mg and Cl-(Na + K) values in epm% (Eid et al., 2024a, 2024b; Sulin, 1946). In the current study, the water samples were of meteoric origin. The water samples of western part of study region fell within shallow meteoric zone has NaHCO_3_ composition and the deep meteoric water samples in eastern part compose of Na_2_SO_4_. To verify that evaporation crystallization/dissolution is the major process influencing water chemistry in the QA, the log ionic ratio of Mg^2+^/Na^+^ versus Ca^2+^/Na^+^ was used (Fig. 5b) (Tlili-Zrelli et al., 2013). The ionic ratio revealed that the majority of samples belonged between the evaporation dissolution and silicate weathering domains, which correspond to the two main mechanisms affecting the chemistry in the QA water. Previous study applied similar log ionic ratio of HCO_3_^−^/Na^+^ versus Ca^2+/^Na^+^ on 105 groundwater samples in Ghana which revealed that the silicate weathering and dissolution of evaporites were the main significant processes governing the water chemistry (Abu et al., 2024).

Ion exchange can be included in the research area because the aquifer composition includes silicate and carbonate minerals. The chloro-alkaline indicators determine whether the water is influenced by direct or reverse ion exchange (CAI-I and CAI-II) by implementing the concentration of Cl^−^, Na^+^, CO_3_^2−^, HCO_3_^−^, SO_4_^2−^, and NO_3_^−^ (Fig. 5c, d). Such indices are a particularly useful tool for demonstrating water–rock interaction by replacing ions like Ca^2+^ and Mg^2+^ with Na^+^ and K^+^ which was utilized by several global studies recently (Boualem & Egbueri, 2024). In the current investigation, the CAI-I and CAI-II results showed that the majority of samples had negative values less than zero, indicating that direct ion exchange is an important mechanism influencing the water chemistry of the Quaternary aquifer. Direct ion exchanges replace calcium and magnesium in water with sodium and potassium in rock as mentioned in the following equations.

$${1}/{\text{2Ca}}^{{{2} + }} - {\text{X }} + {\text{ Na}}^{ + } \to {1}/{\text{2Ca}}^{{{2} + }} + {\text{ Na}}^{ + } - {\text{X}} \;\;\;\; \left( {\text{Reverse ion exchange}} \right)$$

$${\text{Na}}^{ + } - {\text{X}} + {1}/{\text{2Ca}}^{{{2} + }} \to {\text{Na}}^{ + } + {1}/{\text{2Ca}}^{{{2} + }} - {\text{X}} \;\;\;\;\; \left( {\text{Direct Ion exchange}} \right)$$

### Geochemical modeling and ion source detection

**Fig. 6:**
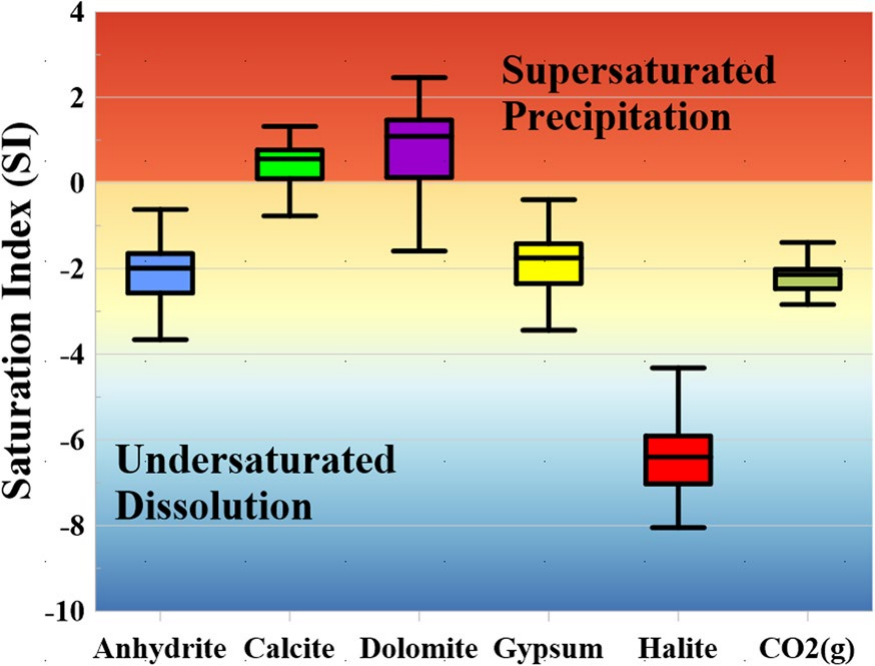
Box plot showing the saturation state and type of minerals extracted from PHREEQC simulation model

The geochemical model was carried out using PHREEQC for identifying the minerals' saturation state, and the association between the ions and the saturation index (SI) may identify the primary impact of minerals in the aquifer system that could increase the amount of the ions calcium, magnesium, chloride, and sodium (Fig. 5). The model’s hypotheses are based on the input physical and chemical attributes or parameters, and the output is the saturation index of five minerals. Mineral saturating indicators (SI) were obtained for calcite, gypsum, anhydrite, and dolomite and displayed on a box plot (Fig. 6). To ensure that the simulated minerals are accurate, temperature and pH values measured in the field were used to represent the true state of the aquifer condition, where mineral saturation is sensitive to the physical parameters. In the present research, the saturated salts and minerals contained in water have been determined to identify the type of minerals that may develop and precipitate in the soil, reduce its permeability/infiltration rates, cause a water logging problem, and observe the origin of the chemical variables. The model also supplied the partial pressure of CO_2_, which was found as under-saturated with a negative value, indicating a drop in the aquifer’s recharge amount. The QA water samples had a salt combination assemblage, which included NaCl, Na_2_SO_4_, NaHCO_3_, Mg (HCO_3_)_2_, and Ca (HCO_3_)_2_, as a result of leaching, terrestrial salt dissolution, and cation exchange. The cation exchange activities raised Na^+^ concentrations while decreasing Ca^2+^ and Mg^2+^ concentrations in the solution, resulting in a significant increase in water salinity in the western part of study area. Moreover, the loss of Ca^2+^ reduced the degree of water saturation concerning anhydrite, and gypsum minerals. The saturation states of key minerals revealed that all of the water samples were under-saturated for anhydrite, halite, and gypsum minerals. This means that water can dissolve more of these minerals, increasing its salinity. Previous study revealed that the undersaturation of minerals such as halite, anhydrite, and gypsum could be due to low residence time of the meteoric water rock interaction specially in the shallow aquifer (Abu et al., 2024). Most samples are supersaturated in calcite and dolomite, with minimum values of − 0.99 and − 1.59, and maximum values of 1.32, and 2.46, and mean or average value 0.38 and 0.77 respectively (Fig. 6 and Table S6). The amount of precipitation of the aforementioned minerals in the soil’s composition can reduce infiltration, cause waterlogging, and reduce plant productivity. Calcium fertilizers should not be used in the research area to avoid deterioration of the physical and chemical structures.

The saturation of calcite and dolomite change from supersaturated in western study area to under saturated in the eastern part close to the Nile River, which indicate that there can be mixing between the Nile River water and Quaternary aquifer water. The over extraction of water from the QA close to the Nile River could create cone of depression in the water table and permit to River water to charge the shallow aquifer and dilute the water and decrease the salinity, hardness and saturation state of calcite and dolomite. These findings confirm previous study was performed on the Nile River in this area showing its undersaturation with calcite and dolomite minerals with water type Ca–Mg–HCO_3_. In the current study showed the water samples close to the Nile River have similar signature to the water from the Nile water in the water type, which indicate that the Nile River in the study area recharge the QA.

### Ionic ratio and ion sources

**Fig. 7:**
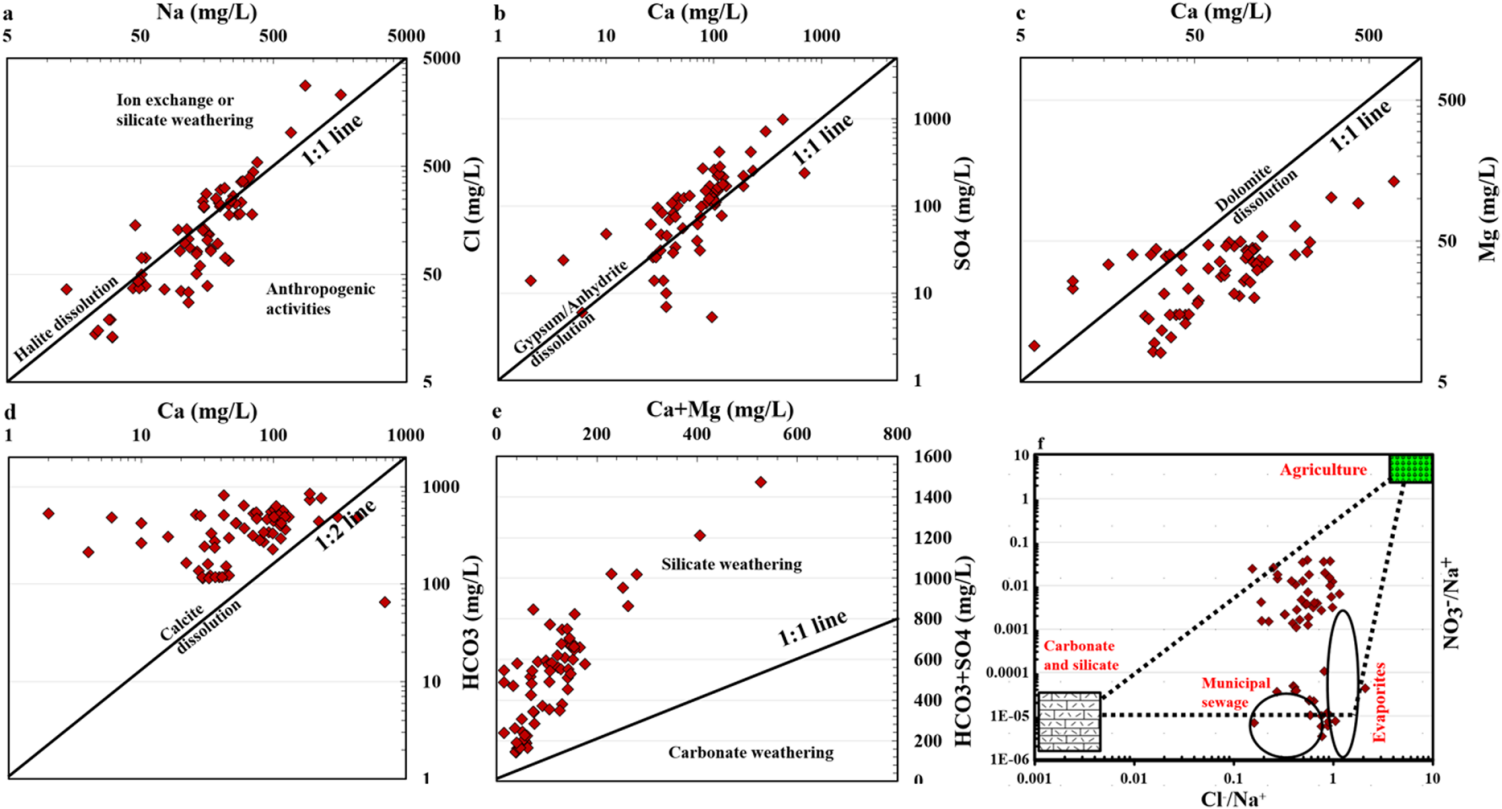
The ionic ratio between the major ions in the flood plain aquifer (QA)

The statistical examination of the associations and ratios of various the primary ions was used to understand the fundamental mechanisms influencing the chemical makeup of groundwater in the research area (Fig. 6). The linear graph of Na^+^ versus Cl^−^ (Fig. 7a) revealed a balanced presence of these ions in most QA samples scattered along 1:1 line, with a strong correlation (0.29) (Fig. 5s). This link implies that halite dissolution contributes significantly to sodium and chloride ions, indicating a Na–Cl water type in the western part of investigated study area. Significant portion of samples shifted to the right of the 1:1 line (weak correlation), indicating additional Na^+^ sources from silicate minerals weathering, ion exchange, and anthropogenic activities leaching from topsoil due to irrigation water return back to the aquifer with high content of Na^+^ (Fig. 3s). Fewer measurements shifted to the left, indicating Na+ removal by reverse ion exchange or an additional source of Cl^−^, including the sylvite minerals, as confirmed by a high correlation (0.7) of Cl^−^ with K^+^ and/or anthropogenic activities (Fig. 3s).

In the case of dominating gypsum disintegration or dissolution, the amount of calcium and sulfates ratio should be one, as observed in certain investigation area samples. Yet, several samples deviation to the right axis from the 1:1 line owing to excessive Ca^2+^ (Fig. 7b), indicating a greater influence of another source of Ca ion rather than gypsum disolution (carbonate and/or silicate weathering). Samples deviated to the left axis of the 1:1 line indicate that SO_4_^2−^ comes from sources other than gypsum and anhydrite breakdown, which could be from anthropogenic activities. The ions association of calcium and magnesium had no balance (Fig. 7c) divided samples into two groups: one near to the right axis of the 1:1 line (majority of samples) and few samples deviated to the left axis of the 1:1: line indicating dolomite dissolution is not the primary source of Ca^2+^ and Mg^2+^ and silicate weathering and ion exchange could have more cotribution to increase Mg ions in water. Similarly, the association between calcium and bicarbonates (Ca^2+^ vs HCO_3_^−^) is relatively low in the vast majority of QA samples (0.3). This shows that water is unable to dissolve calcite since there is supersaturation of the mineral. In most samples, the Ca^2+^/HCO_3_^−^ ratio was less than one (Fig. 7d). The increase of HCO_3_^−^ over Ca^2+^ in a shallow aquifer can result from calcite precipitation, which removes Ca^2+^ but leaves HCO_3_^−^ behind, or from direct cation exchange processes that replace Ca^2+^ with other cations like Na^+^. Additionally, the weathering of silicate minerals, microbial activity producing CO_2_, and agricultural practices can contribute to higher HCO_3_^−^ levels relative to Ca^2+^ in groundwater. A linear graph linking sum of Ca^2+^ and Mg^2+^ vs sum of HCO_3_^−^ and SO_4_^2−^ ions helped identify the source of Ca^2+^ and Mg^2+^ in samples (Eid et al., 2024a, 2024b; Gaagai et al., 2023; Gad et al., 2023). Ion exchange, dissolution of gypsum, anhydrite, and precipitation of dolomite, and calcite decreased the Ca^2+^ + Mg^2+^/HCO_3_^−^ + SO_4_^2−^ ratio over one (Fig. 7e), whereas carbonate precipitation and ion exchange removes Ca^2+^ and Mg^2+^ from water.

Anthropogenic activities have significantly impacted the water quality in the study area, primarily due to the discharge of domestic waste water, and agricultural activities. increasing the concentrations of SO_4_^2−^, Cl^−^, and NO_3_^−^. The elevated levels of SO_4_^2−^ and Cl^−^ in water samples are primarily due to evaporite dissolution and agricultural activities, while NO_3_^−^ mainly originates from domestic sewage and agricultural activities.

The ratios of Cl^−^/Na^+^ versus NO_3_^−^/Na^+^ (Kou et al., 2019) ratios were used to characterize the influence of anthropogenic natural activities on water quality. As shown in Fig. 7f, the majority of samples shallow aquifer (QA) specially in the east of investigated area were affected by agricultural activities, while evaporite and municipal activities had significant impact on the western part of the study area samples.

### Principal component and cluster analysis

**Fig. 8:**
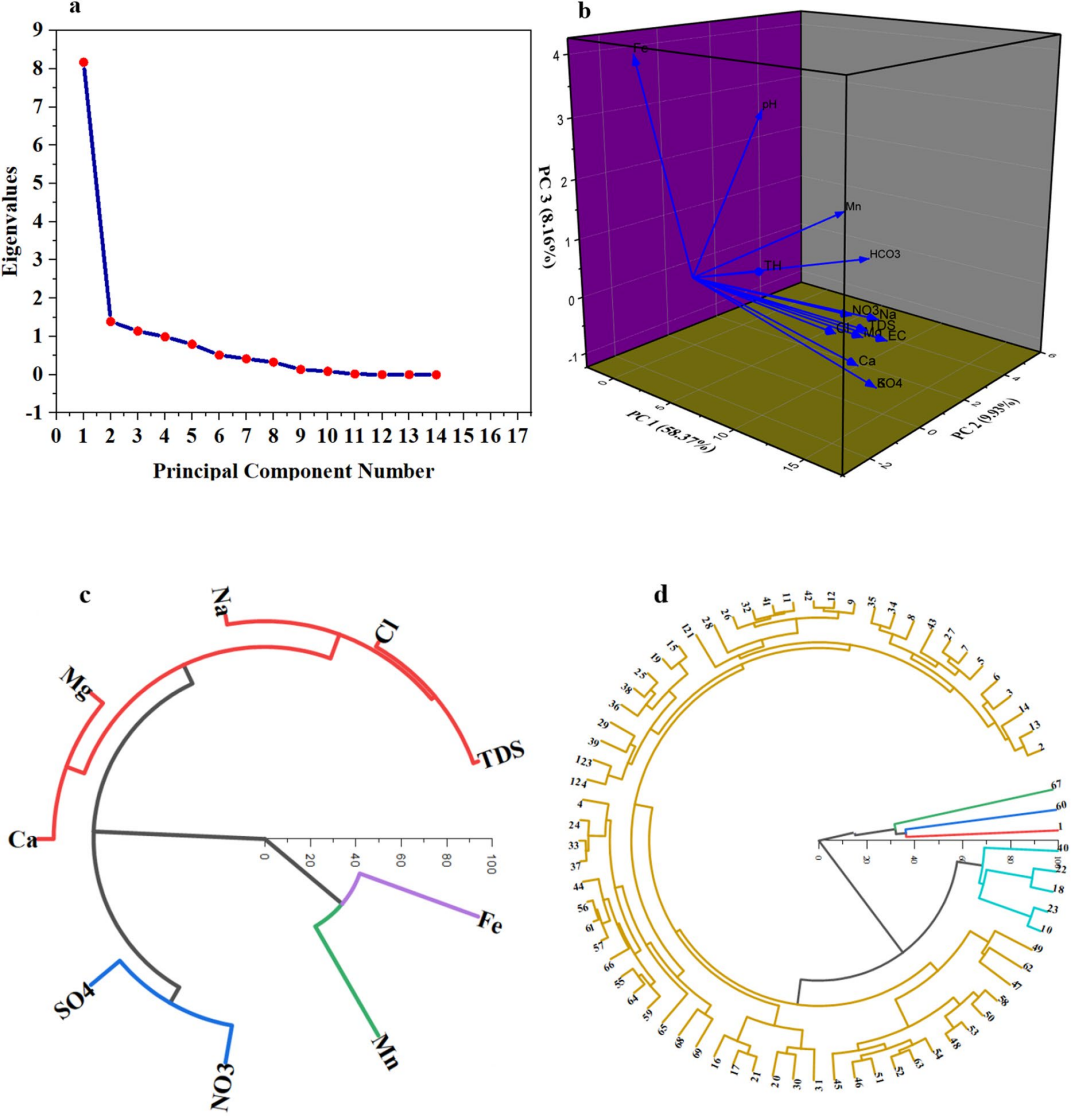
The extracted components from PCA based on scree plot (**a**), PCs in 3D plot (**b**), and cluster analysis using Dendrogram circle showing the correlation between ions (**c**), and samples (**d**)

The Kaiser–Meyer–Olkin analysis (≥ 76.47) and Bartlett’s Sphericity test (*P* < 0.05) validate the suitability of the water quality dataset for PCA, indicating adequate inter-variable relationships. The eigenvalues were higher than 1 (Table 3) which proof the optimum number of components extracted is acceptable for interpretation of the datasets (Fig. 8a).

**Table 3:** The three extracted components (PC1, PC2, and PC3) from PCA using physico-chmical and PTEs parameters

Parameters	PC1	PC2	PC3
TDS	0.335	− 0.090	0.044
pH	0.017	0.364	0.519
EC	0.342	− 0.005	− 0.004
Na^+^	0.336	− 0.033	0.063
K^+^	0.307	0.072	− 0.196
Mg^2+^	0.314	− 0.038	− 0.002
Ca^2+^	0.318	− 0.079	− 0.075
Mn	0.128	0.516	0.188
Fe	0.007	− 0.273	0.754
Cl^−^	0.327	− 0.234	0.069
SO_4_^2−^	0.307	0.072	− 0.196
HCO_3_^−^	0.141	0.632	0.004
NO_3_^−^	0.288	− 0.009	0.041
TH	0.207	− 0.213	0.178
Eigenvalue	8.17	1.39	1.14
Percentage of variance	58.37%	9.93%	8.16%
Cumulative	58.37%	68.31%	76.47%

PC1, accounting for 58.37% of the variance, shows a correlation between TDS, TH, EC, Na^+^, K^+^, Ca^2+^, Cl^−^, SO_4_^2−^, and NO_3_^−^ (Table 3 and Fig. 8b). This suggests that these variables may share a common source or underlying relationship in the context of water quality. PC1 represent the water rock interaction and dissolution process of gypsum and halite minerals are the main reason for increasing the salinity of water in the aquifer system. Similar studies in arid countries such as Algeria and Saudi Arabia applied cluster analysis and PCA for physicochemical parameters and could confirm the high correlation of the above mentioned parameters TDS, TH, EC, Na^+^, K^+^, Ca^2+^, Cl^−^, and SO_4_^2−^ refers to water rock interaction and mineral dissolution (gypsum and halite minerals) are the main reason for increasing the salinity of water in the aquifer system (Abba et al., 2023; Boualem & Egbueri, 2024).

PC2, explaining 9.93% of the variance, demonstrates a correlation between HCO_3_^−^ (Table 3 and Fig. 8b) with Mn, implying that these elements may originate from a similar source and/or geological process. PC2 represent the anthropogenic activity source where agricultural practices and irrigation water return back to the aquifer contaminated with fertilizers beside silicate weathering can contribute to higher HCO_3_^−^ levels relative to Ca^2+^ in groundwater. Fertilizers often contain compounds that can lead to the formation of bicarbonates in water through various chemical reactions. Additionally, certain agricultural practices might lead to increased mobilization of manganese from the soil into groundwater. The use of certain types of irrigation water or amendments can affect the redox conditions in soils, potentially increasing the dissolution and mobilization of manganese into groundwater (Nadarajan & Sukumaran, 2021). In areas with heavy industry or mining, manganese can leach into groundwater. Discharge of industrial or domestic wastewater that contains elevated levels of manganese or organic matter can also contribute to elevated manganese levels in groundwater. The organic matter can change the redox conditions, enhancing the release of manganese from sediments (Islam & Mostafa, 2023; Usman et al., 2021) which is the case in the current study.

PC3, contributing 8.16% of the variance, shows Fe standing alone with pH (Table 3 and Fig. 8b), indicating that the concentration of iron in the groundwater is controlled by the acidity/alkalinity of water based on the contribution of geogenic and anthropogenic sources in the different location of investigated study. Increasing the pH value and changing the water from neutral to alkaline condition facilitate the precipitation of iron in the rock matrix and decrease its concentration in water, which is the case in the current study. The PCA tool was effective to analyze the main component and mechanisms could control the water chemistry and confirm the previous statistics and geochemical model. The solubility of iron in water is strongly dependent on pH. In general, iron is more soluble in acidic conditions (low pH) and precipitates out of solution as iron oxides or hydroxides in more alkaline conditions (higher pH). If the groundwater has a low pH, it can dissolve iron-bearing minerals, increasing the concentration of dissolved iron. Conversely, as pH increases, iron may precipitate out of solution, reducing its concentration in the water (Schwertmann, 1991). Improper disposal of industrial or domestic wastewater can introduce substances that affect both pH and iron levels in groundwater. Organic matter and certain chemicals can alter the redox conditions and pH, leading to changes in iron solubility. The breakdown of organic matter in soil and water can produce acids, lowering the pH. This process can also consume oxygen, creating reducing conditions that favor the solubilization of iron (Grybos et al., 2009). Contaminants like iron and manganese can have health implications if present in drinking water at high concentrations. The study’s results can inform public health strategies to monitor and mitigate the exposure to these elements, thereby contributing to SDG 3 (Abioye & Perera, 2019). This study highlights the importance of enhancing water quality to support the goals of SDG 6.1, which aims for universal and fair access to safe drinking water, and SDG 3, which focuses on ensuring good health and well-being, in Egypt.

The Ward’s method and dendrogram analysis revealed the formation of four distinct clusters based on the similarities in the water quality variables. Interpreting a dendrogram from a cluster analysis involves understanding the relationships and similarities between different groundwater quality parameters. A dendrogram is a tree-like diagram that illustrates the arrangement of clusters produced by hierarchical clustering. There are four main clusters (Fig. 8c) extracted from dendrogram as follows;

*Cluster 1 *TDS, Ca^2+^, Mg^2+^, Na^+^, Cl^−^

These parameters are often related to mineral dissolution and ion exchange processes in fresh groundwater. The presence of TDS, Ca^2+^, Mg^2+^, Na^+^, and Cl^−^ together suggests that these ions are likely derived from the weathering of carbonate and silicate minerals.

The integration of the cluster1 with piper plot:

Ca–Mg–HCO_3_ Facies (34%): This is the dominant facies, indicating that the majority of groundwater is influenced by carbonate weathering, contributing Ca and Mg. Mixed Na–Ca–HCO3 Facies (17.4%): This facies represents areas where there is a mix of sodium and calcium bicarbonate, indicating ion exchange processes where Na replaces Ca in the groundwater matrix. Mixed Ca–Mg–Cl/SO_4_ Facies (21.8%): This facies suggests additional sources of Cl^−^ and SO_4_^2−^, possibly from the dissolution of gypsum (CaSO_4_) and halite (NaCl), or from agricultural activities.

*Cluster 2 *SO_4_^2−^and NO_3_^−^

The grouping of sulfate and nitrate suggests influence from agricultural activities, such as the use of fertilizers, or natural oxidation of sulfide minerals and nitrification processes. Mixed Ca–Mg–Cl/SO_4_ Facies (21.8%): This facies likely reflects areas with higher sulfate concentrations, consistent with the Cluster 2 parameters. Ca–Mg–HCO_3_ Facies (34%) and Mixed Na–Ca–HCO_3_ Facies (17.4%): These facies might show lower concentrations of SO_4_^2−^ and NO_3_^−^, indicating less influence from agricultural runoff compared to the mixed Ca–Mg–Cl/SO_4_ facies.

*Cluster 3* Fe

Iron appearing as a separate cluster indicates unique geochemical conditions, such as redox reactions where iron is mobilized under reducing conditions. Fe is not directly represented on the Piper plot but is crucial for understanding the redox conditions and potential contamination sources in the groundwater. Areas with high Fe might overlap with any of the facies but are more indicative of local reducing conditions or natural iron sources. However, in the study area the Fe concentration is very low in most samples due to oxidizing environment as well as the high concentration comes from anthropogenic activities.

*Cluster 4* Mn

Similar to Fe, manganese clustering separately suggests specific redox conditions or geological sources influencing Mn concentrations. Cluster 4 represent the anthropogenic activity source where agricultural practices and irrigation water return back to the aquifer contaminated with fertilizers.

The cluster analyses of the water samples (Fig. 8d) based on measured parameters divided the samples to 5 clusters. The largest cluster (Cluster 1), containing 61 samples, represents the typical groundwater chemistry of the quaternary aquifer, influenced by common processes like carbonate dissolution and ion exchange, encompassing various hydrochemical facies. Cluster 2, located in the south, likely reflects distinct characteristics with potential agricultural impacts, fitting mainly into the Ca–Mg–HCO_3_ facies. Cluster 3, in the northwest, represents a unique sample potentially influenced by localized geological or contamination factors. Clusters 4 and 5, with samples in the central and southwestern parts of the study area, respectively, indicate unique local conditions affecting groundwater chemistry. These clusters highlight the regional variability in groundwater quality, providing insights into specific local influences and broader geochemical processes. These insights can guide targeted investigations and management strategies to address any localized contamination or to understand the regional groundwater dynamics better.

### Groundwater suitability for drinking using WQI

**Fig. 9:**
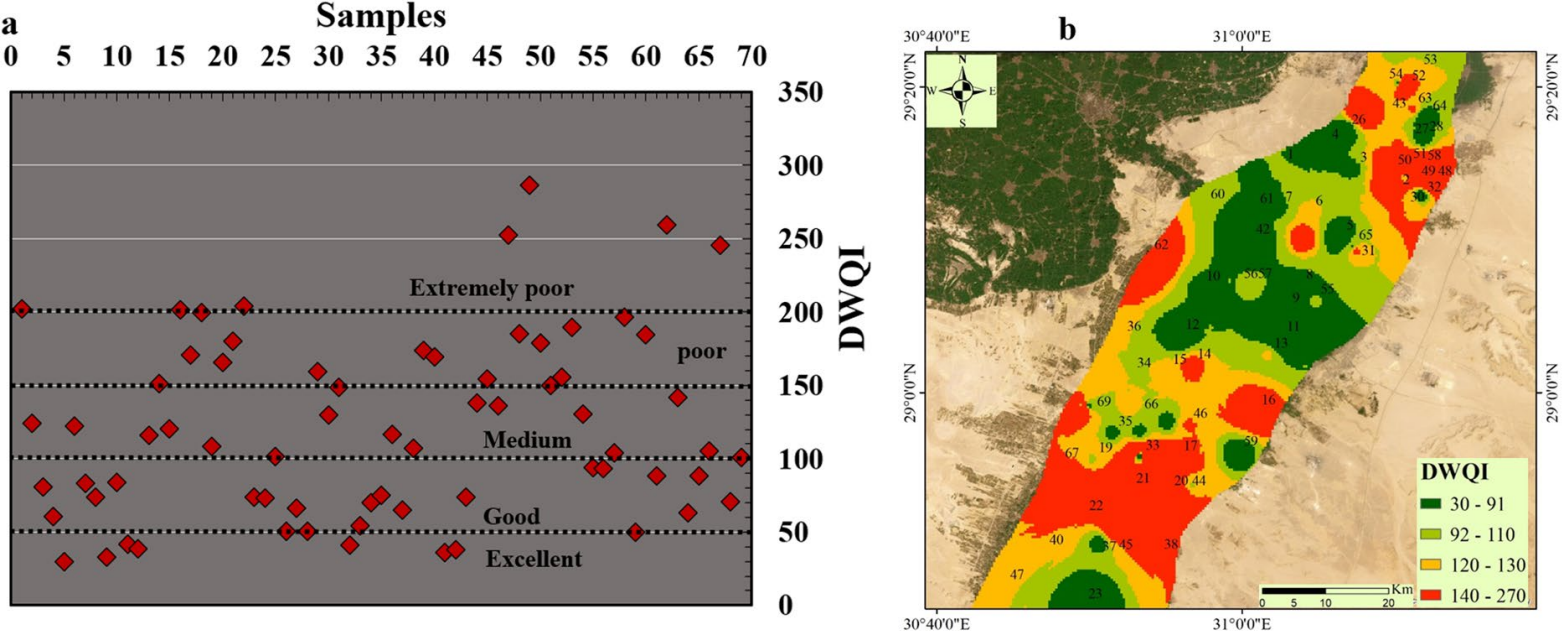
The calculated WQI and plotting its values for all samples (**a**) and WQI distribution map in Beni-Suef area (**b**)

The water quality index for consumption (drinking) purposes based on WQI could be classifies into four main quality type (extremely poor, poor, medium or intermediate good, and excellent quality) according to the calculated values. The range of this classification is > 200, 150–200, 100–150, 50–100, and 0–50 for extremely poor, poor, medium or intermediate good, and excellent quality respectively. In the current study, the WQI ranges from 29.57 to 286.3 with an average value 121.45. Figure 9a classifies water based on WQI values, indicating that 10.14% of water samples fell into the extremely poor category (WQI > 200) including water samples located in north west and south west of study area (S1, S16, S22, S47, S62, and S67), (Fig. 9b). The results showed that 21.7% of samples had WQI values between 150 and 200 which fell within poor quality range represented by 15 samples. The WQI value in 26% of samples raged from 100 to 150 including 18 samples which indicates medium quality category. The rest of samples (42% of samples) fell within good to excellent quality represented by 29 samples. The distribution of the WQI values (Fig. 9b) showed the most deteriorated locations in the north east and south of the investigated area which need more attention from the decision makers to make further treatment of water in this location to avoid any health risk could develop from contaminated water. Deterioration of the water quality close to the Nile River could be because of mixing contaminated water of the Nile River with groundwater of QA where all industrial and agricultural drainages discharge water to the Nile River. The current findings demonstrated that the groundwater of the QA aquifer in the central region is the most appropriate for drinking based WQI, while the other parts of the investigated area need further treatment and suitable management to prevent any health risks regarding the water quality.

### Heavy metal pollution index (HPI)

The utilization of the heavy metal pollution model (HPI) constitutes a fundamental approach for comprehensively calculating the pollution levels within surface and groundwater environments. The utilization of this model facilitates the evaluation of PTEs impacts on freshwater quality, thereby enabling effective monitoring and management strategies to mitigate potential risk linked to with PTEs exposure. Water quality is categorized as excellent (HPI < 15), good to intermediate (15 < HPI < 30), poor (76 > HPI > 51), very poor (76 < HPI < 100), and unsuitable (HPI > 100). Notably, the mean HPI value during the period of observation recorded an average of 35, demonstrating a range spanning from 0.37 to 139. These results underscore the substantial presence of heavy metal contaminants in the groundwater samples collected within the study period.

According to above mentioned classification, nearly 4.34% of the samples fell within the unsuitable water quality category (HPI > 100), indicating high levels of heavy metal contamination in three locations (S47, S49, and S62) in the north, south and west of the study area. Approximately 11.6% of the samples fell within very poor category (100 < HPI < 76), indicating relatively high levels of heavy metal contamination in eight locations (S16, S18, S21, S22, S48, S50, S53, and S58) in the north, south and west of the study area. The current results showed about 13% of the samples fell within poor category (76 > HPI > 51), indicating the presence of contamination of water with PTEs. Therefore, there are several important considerations regarding the underlying factors driving heavy metal pollution in the study area. Environmental factors such as industrial activities, urbanization, and agricultural practices may contribute to the influx of heavy metals into groundwater bodies, exacerbating pollution levels.

**Fig. 10:**
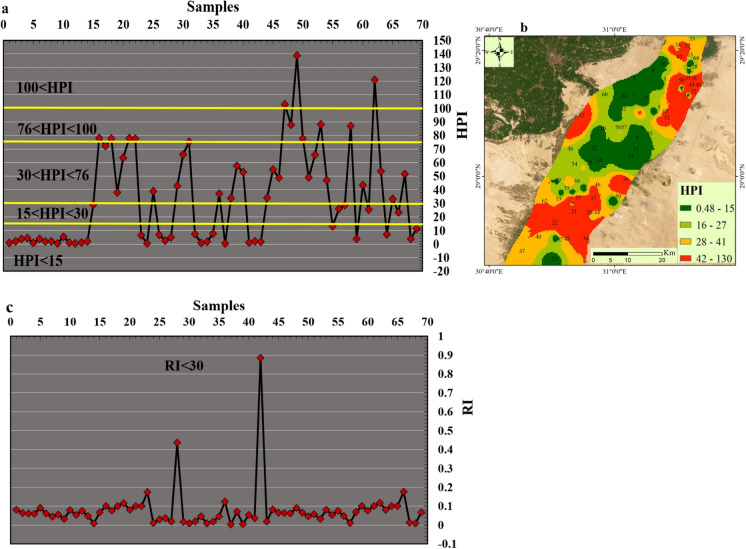
Scatter plot of the PTEs indices including HPI (**a**), HPI distribution map (**b**) and ecological risk values calculated from the PTEs with each sample (**c**) in Beni-Suef

Additionally, natural processes such as weathering and erosion can also influence the mobilization and transport of heavy metal contaminants, further exacerbating the issue. Comprehensive measures are essential to address these pollution levels effectively and protect the environment for current and future generations. The water quality in QA regarding PTEs changed in the rest of samples to be between Excellent and good category with HPI value below 15 in 46.4% of samples and between 15 and 30 in 7.2% of water samples (Fig. 10a). The distribution of the HPI values (Fig. 10b) showed the most deteriorated locations in the north east and south of the investigated area which need more attention from the decision makers to make further treatment of water in this location to avoid any health risk could develop from contaminated water. Deterioration of the water quality close to the Nile River could be because of mixing contaminated water of the Nile River with groundwater of QA where all industrial and agricultural drainages discharge water to the Nile River. The current findings demonstrated that the groundwater of the QA aquifer in the central region is the most appropriate for drinking based HPI, while the other parts of the investigated area need further treatment and suitable management to prevent any health risks regarding the water quality.

### Ecological risk index (RI)

The Possible Ecological Risks Indicator, stands as a commonly acknowledged approach utilized to measure the degree of PTEs pollution and its plausible ramifications on both sedimentary and aquatic environments (Hakanson, 1980; Saeed et al., 2023a). This indicator considers various parameters, encompassing heavy metal concentrations, their toxicological and ecological effects, as well as greater environmental effects. This work focused on the ecological risk indicator (RI) of PTEs (Fig. 10c) in groundwater of Ben-Suef Quaternary aquifer. The computed average RI value for the investigated samples stood at 0.09, ranging from 0.004 to 0.88. These observations indicate that the groundwater of the QA in all location of the investigated area does not have any ecological risk regarding PTEs with very low RI value (RI < 30). However, the investigated area is characterized by considerable agricultural and residential sectors proximate to the basin and its tributaries and could contribute to the accrual of heightened metals amounts in sedimentary deposits, subsequently infiltrating into the study area, there is still no potential risk could be significant.

### Human health risk assessment

**Fig. 11:**
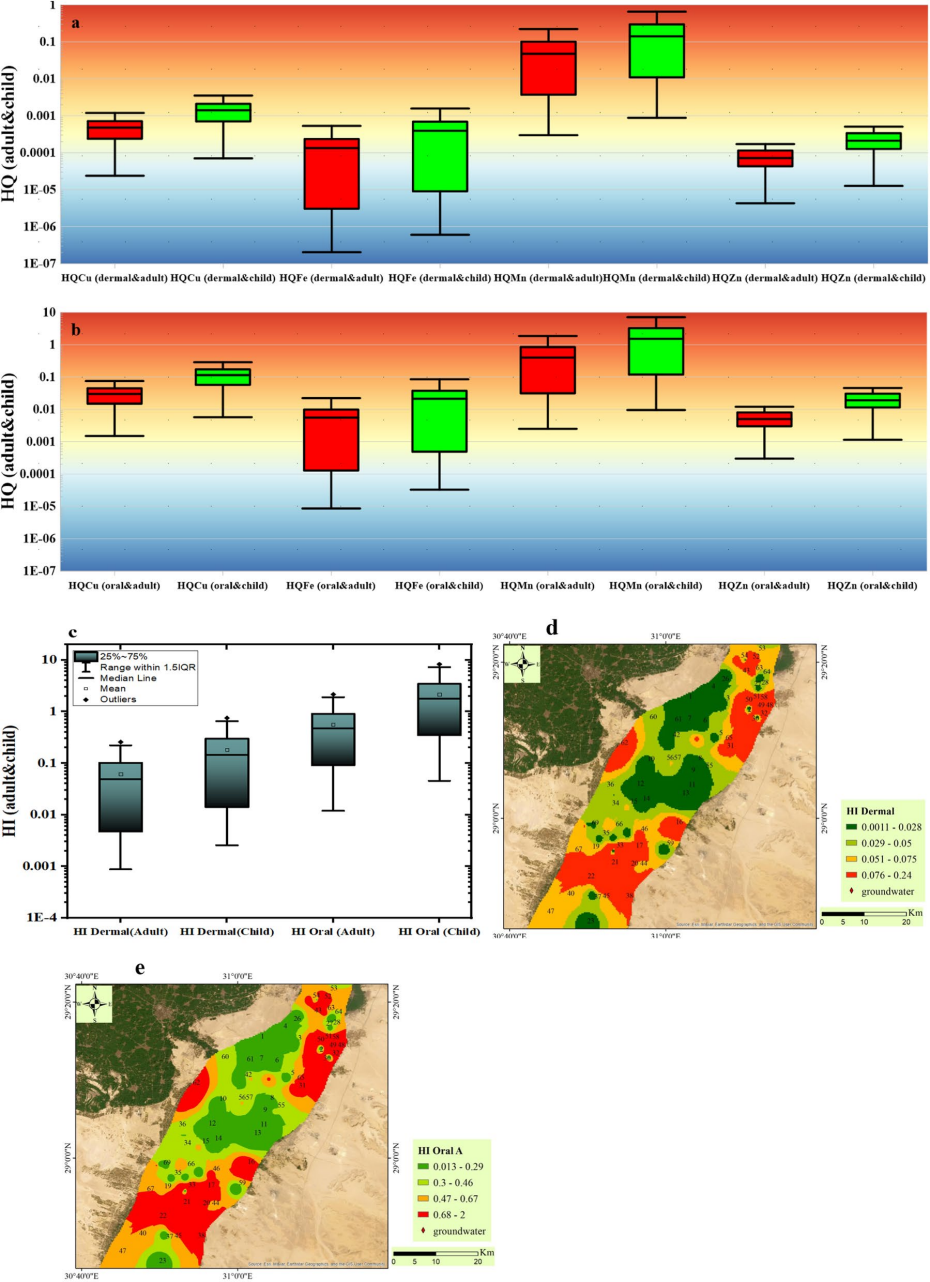
Box plot illustrate the risk indices including HQ dermal (**a**), HQ oral (**b**), HI (**c**), distribution maps of HI dermal (**d**), HI oral in adult and child

The evaluation of non-carcinogenic and carcinogenic risk hazard indices involved the computation of hazard quotients (HQ) for dermal (Fig. 11a) and ingestion (Fig. 11b) absorption pathways. These results unveil the collective health risks posed to both adults and children due to exposure to various heavy metals.

#### Non-carcinogenic health risk

The hazard quotient (HQ) values for dermal exposure to heavy metals, including Mn, Zn, Fe, and Cu were assessed for both adults and children (Table S7).

For adults, the HQ values ranged from 2.0E−07 to 6.3E−04 for Fe, from 3.0E−04 to 2.5E−01 for Mn, from 2.4E−05 to 1.9E−03 for Cu, and from 4.2E−06 to 1.3E−03 for Zn. Meanwhile, children exhibited higher HQ values compared adult across all metals, with ranges from 6.0E−07 to 1.9E−03 for Fe, from 8.8E−04 to 7.5E−01 for Mn, from 7.0E−05 to 5.6E−03 for Cu, and from 1.2E−05 to 3.7E−03 for Zn. The current results showed that the dermal exposure of PTEs (Cu, Zn, Fe, and Mn) in the groundwater of QA does not threaten the health or the skin of the individuals in Beni-Suef area (adult and child).

On the other hand, the hazard quotient (HQ) values for oral exposure to heavy metals Fe, Mn, Cu, Zn were assessed for two age groups (adults and child) (Table S8). In adults, HQ values for Fe ranged from 8.6E−6 to 2.7E−02, while for children, they varied from 3.3E−05 to 1.0E−01. For Mn, adults exhibited HQ values ranging from 2.5E−03 to 2.1, whereas children displayed values between 9.6E−03 and 8.2. The Cu showed HQ values ranging from 1.5E−03 to 1.2E−01 in adults and from 5.8E−03 to 4.6E−01 in children. zinc exhibited HQ values ranging from 3.0E−04 to 8.8E−02 in adults and from 1.2E−03 to 3.4E−01 in children. The current findings revealed that the children and adults are more vulnerable to ingestion exposure of PTEs more than dermal exposure especially for Mn metals in the groundwater of QA in significant number of samples. In case of adults group, 21.7% of samples located in the north and south part of study area has high risk regarding Mn metal exposure with high value of HQ (HQ > 1), while the worst case in the children group which showed high risk in more locations (53.6% of samples) with HQ value greater than one. It was confirmed before from the multivariate statistical analysis between ions that main contributor or source of elevated manganese in groundwater is anthropogenic activities. It was noted also that the most toxic metal responsible for water contamination in the QA and could cause health risk specifically in the north and south part of Beni-Suef region is manganese metal. The Hazard quotient value can explain the health risk of every toxic metal separately, while the sum of HQ for all PTEs could give general risk by combining all metals together.

Additionally, HI for dermal exposure showed values in adults ranging from 9.0E−04 to 0.255 and in children from 0.003 to 0.752. These findings highlight the safety of skin contact with the groundwater of QA in the two groups of different age (child and adult) in all the investigated area with very low HI value (HI < 1) (Fig. 11a). However, the HQ level is higher in the north and south part compared to the central study area (Fig. 11d). In comparison, for hazard index (HI) through oral/ingestion, adults exhibited HI values ranging from 0.012 to 2.16, while children showed higher values, ranging from 0.045 to 8.25 (Fig. 11b). In case of adults group, 21.7% of samples located in the north and south part of study area (Fig. 11e) has high risk regarding PTEs exposure with high value of HI (HI > 1), while the worst case in the children group which showed high risk in more locations (55% of samples) with HI value greater than one.

These results underscore varying degrees of health risks connected with heavy metal exposure, with children consistently showing higher HI values across all metals, highlighting the increased vulnerability of children to heavy metal toxicity. Such findings emphasize the critical need for targeted interventions to reduce exposure and protect public health, particularly among children in the north and south part of study area.

#### Monte Carlo simulation approach

Monte Carlo simulation was used to estimate the hazard quotient (HQ) values for oral and dermal exposure to of Fe, Mn, Zn, and Cu, and demonstrate reliable risk by decreasing the uncertainty in the datasets. By using python code and 10,000 epochs or iterations in the concentration of metals and reference standards of EPA, the reliable risk regarding every toxic metal was estimated.

##### Non-carcinogenic risk

**Fig. 12:**
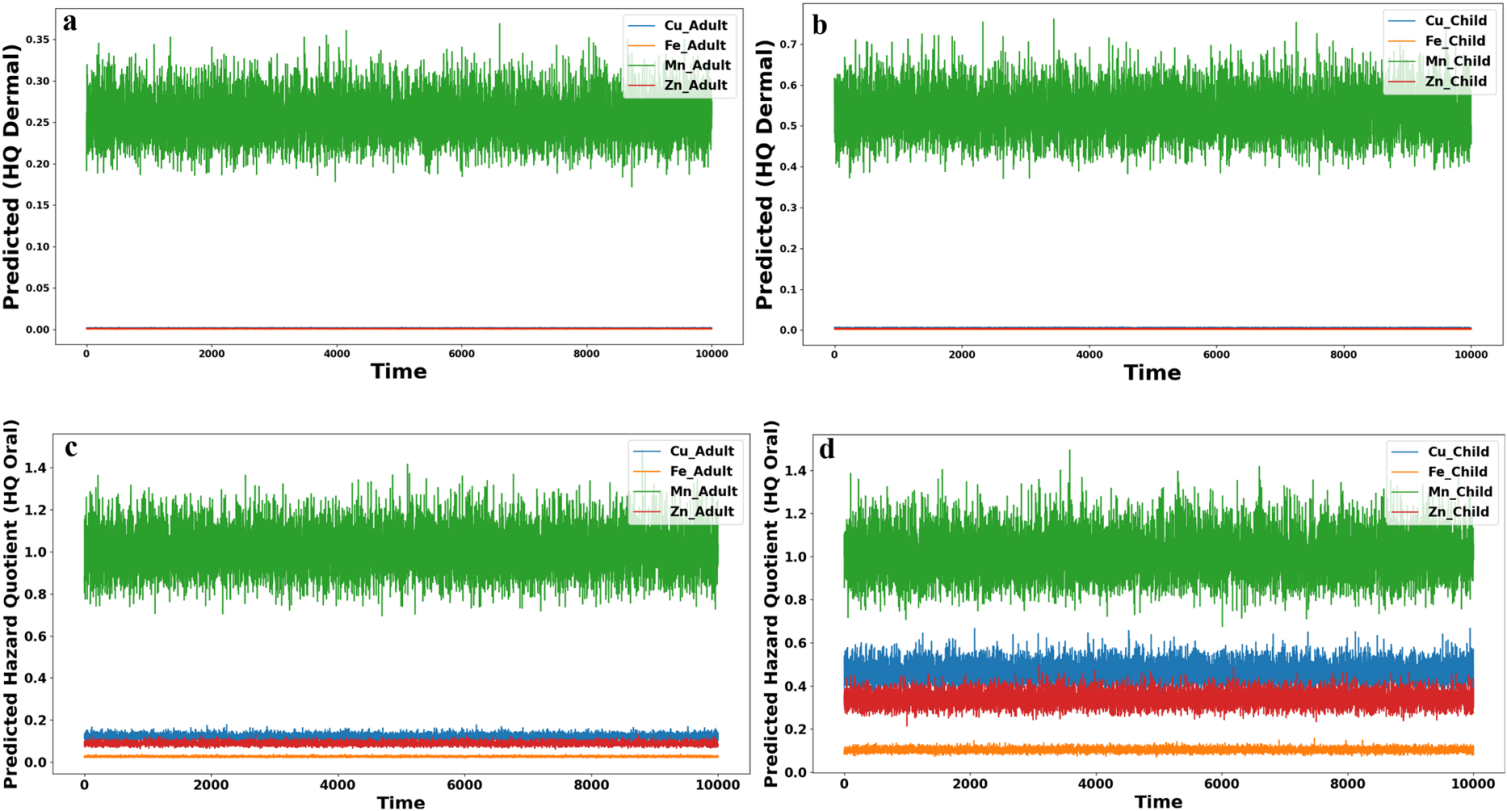
The Hazard quotient simulated from Monte Carlo in all groups with two different ways of exposure

The Monte Carlo simulation results indicated that the predicted dermal hazard quotient (HQ) values for adults for all assessed heavy metals (Fe, Mn, Cu, and Zn) remained below the standard safety threshold (HQ < 1) (Fig. 12a), suggesting a manageable risk level for the adult population. similarly, for children, the dermal HQ values for Fe, Mn, Cu, and Zn did not exceed the threshold limits (HQ < 1) (Fig. 12b), highlighting a neglected health risk and no need for urgent attention to reduce dermal exposure levels to the measured metals in the two groups (adult and child). In contrast, the oral HQ values Cu, Fe, and Zn in both adults and children did not exceed the standard limits (HQ < 1) (Fig. 12c, d), confirm the safety of oral exposure to these metals in the groundwater of the quaternary aquifer. The results of Monte Carlo simulation showed that the health risk of groundwater consumption developed only through oral exposure to Mn metal with predicted HQ value greater than one (HQ > 1) in adult and child. These findings indicate a critical risk associated with oral exposure to manganese. However, it’s important to recognize that risk assessments often rely on conservative assumptions and uncertainties in existing data. Therefore, continuously monitoring exposure levels and updating risk assessments with new information as it becomes available is crucial such as measuring Cd, Cr, and Pb metals to evaluate the carcinogenic risk through different exposure routes.

Application of probabilistic method such as Monte Carlo simulation could confirm the traditional calculation and give more realistic interpretation of the health risk of PTEs in the groundwater of the Quaternary aquifer in Beni-Suef region and could be applied globally in different study area.

#### Limitations, implications, and gaps for future work

The study’s findings on groundwater quality in the Beni-Suef area have significant implications for climate action and several SDGs. By providing a comprehensive understanding of the factors affecting groundwater quality, the research can inform policies and strategies that promote sustainable water management, public health, and environmental protection. Addressing these issues is critical not only for the local population but also as part of broader efforts to achieve sustainable development and resilience in the face of climate change.

Although the current study covers different crucial points integrate between hydrochemistry, ion source and contamination origin with environmental, ecological, and health risk assessment supported with simulation model, there are still some limitations that could be covered in the future work.

Application of stable isotopes and mixing model to detect the reliable pollution source and contribution percentage from the Nile River to the QA or vice versa and the effect of mixing of surface water and groundwater on the water quality. Measuring the carcinogenic elements such as Cd, Cr, and Pb to determine the carcinogenic risk on the human health and provide water management and treatment plan to avoid any health issues. The future work can include also collecting soil samples to detect the accumulation of PTEs in the soil and its effect on the groundwater quality, plant production, and soil fertility.

## Conclusion

This study investigates the drinking water quality and health, ecological, and environmental risks associated with potentially toxic elements (PTEs) in the floodplain Quaternary aquifer (QA) located in Beni-Suef, Egypt. A comprehensive approach was used, including the PHREEQC geochemical model, ionic ratios, and multivariate statistical analysis such as principal component (PCA) and cluster (dendrogram) analysis, to estimate the sources of ions, contamination, and the mixing of Nile water with QA. An advanced method called the integrated weight water quality index (WQI), derived from the entropy method, was applied to determine the suitability of water for drinking. Various indices, such as the Heavy Metal Pollution Index (HPI), ecological Risk Index (RI), Hazard Quotient (HQ), and total Hazard Index (HI), were used to assess the ecological, environmental, and human health risks regarding PTEs in QA. Furthermore, the Monte Carlo method was applied for the probabilistic assessment of non-carcinogenic risks through oral and dermal exposure routes in both adults and children. A GIS tool was used to interpolate all indices in the study area to detect the most deteriorated locations for sustainable management.

The hydrochemical characteristics indicated that the water type/facies were Na–Cl, Ca–Mg–HCO_3_, mixed Na–Ca–HCO_3_, mixed Ca–Mg–Cl/SO_4_, and Na–HCO_3_. The mechanisms controlling water quality are carbonate dissolution, direct ion exchange, silicate weathering, and evaporation/crystallization. The sources of contamination with NO_3_- and PTEs in the north and south parts of the study area are agricultural activities, irrigation water returns, municipal waste, and evaporites.

Based on WQI values, 10.14% of water samples fell into the extremely poor category (WQI > 200), 21.7% of samples had WQI values between 150 and 200, falling within the poor-quality range, and 26% ranged from 100 to 150, indicating a medium quality category. The rest of the samples (42%) fell within the good to excellent quality range.

Water quality regarding PTEs fell between excellent and good, with HPI values below 15 in 46.4% of samples and between 15 and 30 in 7.2% of water samples in the central study area, while the north and south are contaminated. The calculated ecological Risk Index (RI) in all samples was below the threshold limit (RI < 30), confirming the water’s safety and lack of ecological risk from PTEs.

The application of Monte Carlo simulation revealed no health risks for children and adults through skin contact, but a high risk from oral exposure (HQ predicted > 1) to manganese (Mn). The results demonstrated that the north (El-Wasta city) and south (El-Fashn city) parts of the study area face different contamination challenges based on PTEs, WQI, HPI, HQ, and HI, necessitating further treatment and management of water before consumption.

## Supplementary Information

Below is the link to the electronic supplementary material.

Supplementary file1 (DOCX 55 kb)

## Data Availability

The datasets utilized and/or analyzed during the current study are available upon request from the corresponding author.
