# Correction: Comprehensive approach integrating water quality index and toxic element analysis for environmental and health risk assessment enhanced by simulation techniques

**DOI:** 10.1007/s10653-024-02225-7

**Published:** 2024-09-16

**Authors:** Mohamed Hamdy Eid, Mahmoud Awad, Essam A. Mohamed, Tamer Nassar, Mostafa R. Abukhadra, Ahmed M. El-Sherbeeny, Attila Kovács, Péter Szűcs

**Affiliations:** 1 Institute of Environmental Management, Faculty of Earth Science, University of Miskolc, 3515 Miskolc- Egyetemváros, Hungary; 2 Geology Department, Faculty of Science, Beni-Suef University, Beni-Suef, 65211 Egypt; 3 Faculty of Earth Science, Beni-Suef University, Beni-Suef, 62511 Egypt; 4 Geology Department, Faculty of Science, Cairo University, Cairo, Egypt; 5 Industrial Engineering Department, College of Engineering, King Saud University, P.O. Box 800, 11421 Riyadh, Saudi Arabia

**Correction to: Environ Geochem Health (2024) 46:409** 10.1007/s10653-024-02182-1

In the Acknowledgements section of this article, the Researchers Supporting Project number relating to King Saud University, Riyadh, Saudi Arabia was incorrectly given as RSPD2024R804 but should have been RSP2024R133.

The original article has been corrected.

