# Supplementary material for: Comprehensive approach integrating water quality index and toxic element analysis for environmental and health risk assessment enhanced by simulation techniques
Source: Environ Geochem Health. 2024 Aug 31;46(10):409. doi: 10.1007/s10653-024-02182-1 (PMC11365846; doi:10.1007/s10653-024-02182-1)
Supplement: Supplementary file 1 — Supplementary file1 (DOCX 55 kb) [file 10653_2024_2182_MOESM1_ESM.docx]

**Comprehensive Approach Integrating Water Quality Index and Toxic Element Analysis for Environmental and Health Risk Assessment Enhanced by Simulation Techniques**

Table S1. The results of the three samples used with mean and stand deviation values

| Metals | S1 (mg/L) | S2 (mg/L) | S3 (mg/L) | Mean (mg/L) | STD (mg/L) |
| --- | --- | --- | --- | --- | --- |
| Mn | 0.002 | 1.71 | 0.422 | 0.7113 | 0.8321 |
| Fe | 0.002 | 0.62 | 0.152 | 0.258 | 0.3372 |
| Zn | 0.003 | 0.88 | 0.081 | 0.3213 | 0.4807 |
| Cu | 0.002 | 0.16 | 0.049 | 0.0703 | 0.0866 |

Table S2. The accuracy is determined by comparing the concentrations of the certified reference material to the known values.

| **Metals** | **Mean (measured) (mg/L)** | **CRM Value (mg/L)** | **Recovery (%)** |
| --- | --- | --- | --- |
| Mn | 0.422 | 0.4 | 105.5 |
| Fe | 0.152 | 0.15 | 101.3 |
| Zn | 0.081 | 0.08 | 101.3 |
| Cu | 0.049 | 0.05 | 98 |

Table S3. working standards, along with metal correlation coefficients.

| Metal | Operational/working  standards | Equation of calibration | R^2^ | Sample. No |
| --- | --- | --- | --- | --- |
| Cu | 0.25, 1, 2.5, 5, 7.5 | y = 0.0215x−0.0039 | 0.999 | 1 |
| Fe | 0.05, 1, 2.5, 5, 7.5, 10 | y = 0.001x + 0.0009 | 0.999 | 2 |
| Zn | 0.5, 1, 1.5, 2.5, 3 | y = 0.0791x + 0.0019 | 0.999 | 3 |
| Mn | 0.05, 1, 2.5, 5, 7.5, 10 | y = 0.0025x + 0.0018 | 0.999 | 4 |

Table S4. The instrument's working conditions

| Metal | Type of flame | Wave length  (nm) | Current of lamp  (mA) | Slit width  (nm) |
| --- | --- | --- | --- | --- |
| Cu | (Air/acetylene) | 324.5 | 1.5 | 1 |
| Fe |  | 248.3 | 7.0 | 2 |
| Zn |  | 213.9 | 2.0 | 3 |
| Mn |  | 294.9 | 3 | 4 |

**Table S5.** The parameters for the calculation of HQ, HI, and CR

| HM | Cu | Fe | Mn | Zn |
| --- | --- | --- | --- | --- |
| RfD Oral(mg/kg/day) | 0.04 | 0.7 | 0.024 | 0.3 |
| ABS | 0.3 | 0.2 | 0.04 | 0.2 |
| Rfd Dermal (mg/kg/day) | 0.012 | 0.14 | 0.00096 | 0.06 |
| Kp | 0.001 | 0.001 | 0.001 | 0.0006 |
| Si | 3 | 0.3 | 0.05 | 1 |
| ET Adult (h/day) | 0.58 | 0.58 | 0.58 | 0.58 |
| ET Child (h/day) | 1 | 1 | 1 | 1 |
| SA Adult (cm^2^) | 18000 | 18000 | 18000 | 18000 |
| SA Child (cm^2^) | 6600 | 6600 | 6600 | 6600 |
| CF (L/cm^3^) | 0.001 | 0.001 | 0.001 | 0.001 |
| IR Adult (L/day) | 2.2 | 2.2 | 2.2 | 2.2 |
| IR Child (L/day) | 1.8 | 1.8 | 1.8 | 1.8 |
| EF (day/year) | 350 | 350 | 350 | 350 |
| ED Adult (year) | 70 | 70 | 70 | 70 |
| ED Child (year) | 6 | 6 | 6 | 6 |
| BW Adult (kg) | 70 | 70 | 70 | 70 |
| BW Child (kg) | 15 | 15 | 15 | 15 |
| AT Adult (day) | 25550 | 25550 | 25550 | 25550 |
| AT Child (day) | 2190 | 2190 | 2190 | 2190 |
| Background (µg/g) | 30 | 15000 | 500 | 100 |
| T_r_ | 5 | 1 | 1 | 1 |

Table S6. The saturation index (SI) values of different minerals extracted from PHREEQC model

| Samples | Anhydrite | Calcite | Dolomite | Gypsum | Halite | CO2(g) |
| --- | --- | --- | --- | --- | --- | --- |
| 1 | -1.1 | 0.27 | 0.13 | -0.87 | -4.32 | -2.84 |
| 2 | -2.57 | 0.1 | 0.14 | -2.32 | -5.76 | -2.08 |
| 3 | -2.33 | 0.16 | 0.81 | -2.1 | -5.82 | -2.57 |
| 4 | -3.02 | 0.7 | 1.79 | -2.79 | -7.24 | -2.73 |
| 5 | -3.13 | -0.99 | -1.59 | -2.91 | -6.99 | -1.08 |
| 6 | -2.33 | 0.03 | 0.31 | -2.09 | -5.42 | -1.77 |
| 7 | -3.07 | 1.22 | 2.18 | -2.83 | -7.15 | -2.42 |
| 8 | -1.71 | 0.74 | 1.38 | -1.48 | -6.58 | -2.08 |
| 9 | -2.19 | -0.33 | -0.74 | -1.97 | -7.22 | -2.45 |
| 10 | -3.31 | -0.3 | -0.64 | -3.09 | -5.77 | -1.96 |
| 11 | -2.43 | 0.26 | 0.4 | -2.21 | -7.86 | -2.74 |
| 12 | -2.11 | -0.23 | -0.65 | -1.89 | -7.36 | -2.42 |
| 13 | -2.07 | 0.67 | 1.33 | -1.83 | -5.57 | -2.8 |
| 14 | -2.78 | 1.14 | 2.46 | -2.55 | -5.82 | -2.47 |
| 15 | -3.14 | 0.67 | 1.81 | -2.91 | -7.03 | -2.45 |
| 16 | -3.07 | 0.24 | 1.18 | -2.84 | -5.92 | -2.62 |
| 17 | -5.23 | 0.74 | 1.57 | -5.01 | -7.03 | -2.36 |
| 18 | -5.78 | 0.32 | 1.28 | -5.55 | -6.49 | -2.77 |
| 19 | -5.62 | -0.1 | 0.41 | -5.4 | -6.76 | -2.6 |
| 20 | -4.12 | 0.22 | 0.98 | -3.9 | -6.4 | -2.65 |
| 21 | -5.93 | 0.13 | 1.04 | -5.71 | -6.67 | -2.81 |
| 22 | -4.23 | -0.69 | -0.22 | -4.01 | -6.4 | -2.1 |
| 23 | -3.66 | -0.77 | -0.75 | -3.44 | -5.96 | -2.49 |
| 24 | -1.65 | 0.85 | 1.65 | -1.42 | -6.52 | -2.2 |
| 25 | -2.19 | -0.26 | -0.35 | -1.98 | -7.17 | -2.49 |
| 26 | -1.99 | -0.42 | -1.07 | -1.75 | -7.22 | -2.37 |
| 27 | -2.29 | 0.7 | 1.44 | -2.06 | -7.32 | -2.47 |
| 28 | -2.72 | -0.24 | -0.49 | -2.48 | -7.94 | -2.61 |
| 29 | -1.49 | 0.57 | 1.13 | -1.26 | -5.98 | -2.07 |
| 30 | -2.63 | -0.1 | -0.35 | -2.41 | -8.05 | -2.75 |
| 31 | -2.66 | -0.19 | -0.58 | -2.44 | -8 | -2.64 |
| 32 | -2.39 | -0.57 | -1.23 | -2.17 | -7.81 | -2.25 |
| 33 | -1.87 | 0.36 | 0.78 | -1.64 | -6.06 | -2.23 |
| 34 | -1.55 | 0.32 | 0.77 | -1.33 | -5.97 | -2.21 |
| 35 | -1.65 | 0.37 | 0.8 | -1.42 | -6.44 | -2.02 |
| 36 | -1.74 | 0.14 | 0.04 | -1.51 | -5.94 | -2.1 |
| 37 | -1.76 | 0.76 | 1.47 | -1.53 | -6.11 | -2.44 |
| 38 | -1.93 | 0.14 | 0.2 | -1.72 | -6.81 | -1.72 |
| 39 | -1.97 | 0.6 | 1.3 | -1.73 | -6.44 | -1.97 |
| 40 | -1.3 | 0.02 | -0.06 | -1.07 | -5.52 | -1.58 |
| 41 | -2.36 | -0.42 | -1.04 | -2.13 | -7.82 | -2.34 |
| 42 | -2 | -0.4 | -0.9 | -1.78 | -7.26 | -2.36 |
| 43 | -1.79 | 0.56 | 1.09 | -1.57 | -6.59 | -1.88 |
| 44 | -2.13 | 0.73 | 1.44 | -1.92 | -6.68 | -2 |
| 45 | -2.41 | 0.79 | 1.42 | -2.16 | -6.99 | -2.17 |
| 46 | -1.53 | 0.67 | 1.24 | -1.29 | -5.8 | -2.1 |
| 47 | -1.38 | 0.92 | 1.74 | -1.17 | -5.64 | -1.63 |
| 48 | -1.82 | 0.53 | 0.97 | -1.59 | -6.78 | -1.67 |
| 49 | -1.47 | 1.04 | 1.72 | -1.23 | -5.86 | -1.68 |
| 50 | -1.55 | 0.81 | 1.38 | -1.32 | -5.91 | -2.05 |
| 51 | -1.98 | 0.81 | 1.84 | -1.75 | -5.91 | -2.14 |
| 52 | -1.65 | 0.72 | 1.27 | -1.42 | -6.33 | -1.94 |
| 53 | -1.84 | 1.09 | 2.15 | -1.68 | -6.6 | -1.91 |
| 54 | -1.65 | 0.79 | 1.51 | -1.42 | -6.33 | -2.05 |
| 55 | -1.78 | 1.32 | 2.42 | -1.57 | -6.36 | -2.34 |
| 56 | -1.76 | 0.65 | 1 | -1.55 | -6.39 | -1.97 |
| 57 | -1.58 | 0.82 | 1.24 | -1.36 | -6.46 | -2.04 |
| 58 | -1.48 | 0.81 | 1.55 | -1.26 | -5.87 | -2.13 |
| 59 | -2.25 | 0.2 | 0.26 | -2.02 | -7.09 | -1.81 |
| 60 | -0.62 | 0.93 | 1.49 | -0.39 | -4.84 | -1.93 |
| 61 | -1.69 | 0.56 | 0.85 | -1.47 | -6.3 | -2.21 |
| 62 | -1.07 | 0.95 | 1.52 | -0.85 | -5.33 | -2.13 |
| 63 | -1.52 | 0.9 | 1.61 | -1.32 | -6.35 | -2.04 |
| 64 | -2.03 | 0.7 | 1.38 | -1.81 | -6.6 | -2.06 |
| 65 | -2.56 | 0.1 | -0.02 | -2.35 | -7.95 | -2.59 |
| 66 | -1.45 | 0.36 | 0.5 | -1.22 | -6.1 | -2.09 |
| 67 | -0.95 | 1.03 | 1.96 | -0.74 | -4.16 | -2.11 |
| 68 | -1.39 | 0.77 | 1.35 | -1.18 | -6.01 | -2.11 |
| 69 | -1.26 | 0.72 | 1.09 | -1.03 | -5.61 | -1.39 |

Table S7. The HQ dermal values in both child and adult

| Samples | **HQ_Cu_**  **(dermal&adult)** | **HQ_Cu_**  **(dermal&child)** | **HQ_Fe_**  **(dermal&adult)** | **HQ_Fe_**  **(dermal&child)** | **HQ_Mn_**  **(dermal&adult)** | **HQ_Mn_**  **(dermal&child)** | **HQ_Zn_**  **(dermal&adult)** | **HQ_Zn_**  **(dermal&child)** |
| --- | --- | --- | --- | --- | --- | --- | --- | --- |
| 1 | 1.9E-03 | 5.6E-03 | 1.3E-04 | 3.9E-04 | 7.4E-04 | 2.2E-03 | 7.2E-05 | 2.1E-04 |
| 2 | 8.3E-04 | 2.5E-03 | 1.9E-04 | 5.7E-04 | 3.0E-03 | 8.8E-03 | 7.2E-05 | 2.1E-04 |
| 3 | 1.2E-04 | 3.5E-04 | 2.1E-04 | 6.3E-04 | 6.0E-03 | 1.8E-02 | 8.6E-05 | 2.5E-04 |
| 4 | 1.2E-03 | 3.5E-03 | 7.2E-05 | 2.1E-04 | 7.6E-03 | 2.2E-02 | 5.7E-05 | 1.7E-04 |
| 5 | 6.0E-04 | 1.8E-03 | 1.8E-04 | 5.4E-04 | 7.4E-04 | 2.2E-03 | 1.1E-04 | 3.4E-04 |
| 6 | 1.2E-04 | 3.5E-04 | 2.1E-04 | 6.3E-04 | 6.0E-03 | 1.8E-02 | 8.6E-05 | 2.5E-04 |
| 7 | 2.4E-04 | 7.0E-04 | 2.3E-04 | 6.9E-04 | 3.0E-03 | 8.8E-03 | 5.7E-05 | 1.7E-04 |
| 8 | 3.6E-04 | 1.1E-03 | 1.5E-04 | 4.5E-04 | 3.0E-03 | 8.8E-03 | 7.2E-05 | 2.1E-04 |
| 9 | 7.2E-04 | 2.1E-03 | 9.2E-05 | 2.7E-04 | 3.0E-04 | 8.8E-04 | 2.9E-05 | 8.4E-05 |
| 10 | 1.2E-03 | 3.5E-03 | 5.3E-04 | 1.6E-03 | 7.4E-03 | 2.2E-02 | 8.6E-05 | 2.5E-04 |
| 11 | 2.4E-04 | 7.0E-04 | 1.8E-04 | 5.4E-04 | 1.2E-03 | 3.5E-03 | 7.2E-05 | 2.1E-04 |
| 12 | 1.5E-03 | 4.6E-03 | 1.3E-04 | 3.9E-04 | 6.0E-04 | 1.8E-03 | 7.2E-05 | 2.1E-04 |
| 13 | 1.1E-03 | 3.2E-03 | 1.7E-04 | 5.1E-04 | 1.2E-03 | 3.5E-03 | 4.3E-05 | 1.3E-04 |
| 14 | 1.2E-04 | 3.5E-04 | 1.1E-04 | 3.3E-04 | 3.0E-03 | 8.8E-03 | 1.1E-05 | 3.4E-05 |
| 15 | 4.8E-04 | 1.4E-03 | 3.2E-04 | 9.3E-04 | 5.2E-02 | 1.5E-01 | 8.6E-05 | 2.5E-04 |
| 16 | 6.0E-04 | 1.8E-03 | 3.8E-04 | 1.1E-03 | 1.4E-01 | 4.2E-01 | 1.3E-04 | 3.8E-04 |
| 17 | 3.6E-04 | 1.1E-03 | 3.4E-04 | 9.9E-04 | 1.3E-01 | 3.9E-01 | 1.0E-04 | 3.0E-04 |
| 18 | 1.2E-03 | 3.5E-03 | 6.3E-04 | 1.9E-03 | 1.4E-01 | 4.1E-01 | 1.1E-04 | 3.4E-04 |
| 19 | 3.6E-04 | 1.1E-03 | 3.1E-04 | 9.0E-04 | 6.9E-02 | 2.0E-01 | 1.6E-04 | 4.6E-04 |
| 20 | 2.4E-05 | 7.0E-05 | 1.2E-04 | 3.6E-04 | 1.2E-01 | 3.4E-01 | 1.1E-04 | 3.4E-04 |
| 21 | 6.0E-04 | 1.8E-03 | 3.8E-04 | 1.1E-03 | 1.4E-01 | 4.2E-01 | 1.3E-04 | 3.8E-04 |
| 22 | 1.2E-03 | 3.5E-03 | 6.3E-04 | 1.9E-03 | 1.4E-01 | 4.1E-01 | 1.1E-04 | 3.4E-04 |
| 23 | 4.2E-04 | 1.2E-03 | 4.8E-04 | 1.4E-03 | 1.0E-02 | 3.0E-02 | 2.4E-04 | 7.1E-04 |
| 24 | 1.1E-04 | 3.2E-04 | 3.1E-06 | 9.0E-06 | 7.4E-04 | 2.2E-03 | 1.4E-05 | 4.2E-05 |
| 25 | 7.9E-04 | 2.3E-03 | 2.7E-04 | 8.0E-04 | 7.0E-02 | 2.1E-01 | 2.3E-05 | 6.8E-05 |
| 26 | 6.6E-04 | 1.9E-03 | 2.2E-04 | 6.5E-04 | 1.1E-02 | 3.4E-02 | 3.7E-05 | 1.1E-04 |
| 27 | 6.1E-04 | 1.8E-03 | 2.6E-04 | 7.5E-04 | 3.7E-03 | 1.1E-02 | 1.3E-05 | 3.8E-05 |
| 28 | 6.3E-04 | 1.9E-03 | 3.2E-04 | 9.4E-04 | 7.7E-03 | 2.3E-02 | 6.1E-04 | 1.8E-03 |
| 29 | 6.2E-04 | 1.8E-03 | 1.9E-04 | 5.5E-04 | 7.8E-02 | 2.3E-01 | 8.6E-06 | 2.5E-05 |
| 30 | 1.4E-04 | 4.2E-04 | 8.7E-05 | 2.6E-04 | 1.2E-01 | 3.6E-01 | 7.2E-06 | 2.1E-05 |
| 31 | 6.1E-04 | 1.8E-03 | 2.3E-04 | 6.9E-04 | 1.4E-01 | 4.0E-01 | 1.1E-05 | 3.4E-05 |
| 32 | 6.4E-04 | 1.9E-03 | 1.7E-04 | 4.9E-04 | 1.3E-02 | 3.9E-02 | 5.1E-05 | 1.5E-04 |
| 33 | 6.0E-05 | 1.8E-04 | 6.1E-06 | 1.8E-05 | 1.5E-03 | 4.4E-03 | 1.1E-05 | 3.4E-05 |
| 34 | 4.2E-04 | 1.2E-03 | 2.1E-04 | 6.1E-04 | 2.2E-03 | 6.6E-03 | 1.9E-05 | 5.5E-05 |
| 35 | 5.7E-04 | 1.7E-03 | 2.0E-04 | 5.9E-04 | 1.3E-02 | 3.9E-02 | 5.1E-05 | 1.5E-04 |
| 36 | 2.4E-04 | 7.0E-04 | 2.6E-04 | 7.5E-04 | 6.7E-02 | 2.0E-01 | 1.7E-04 | 5.1E-04 |
| 37 | 6.0E-05 | 1.8E-04 | 7.2E-06 | 2.1E-05 | 8.9E-04 | 2.6E-03 | 4.3E-06 | 1.3E-05 |
| 38 | 7.2E-05 | 2.1E-04 | 2.7E-04 | 7.8E-04 | 6.1E-02 | 1.8E-01 | 1.0E-04 | 3.0E-04 |
| 39 | 2.4E-04 | 7.0E-04 | 2.3E-04 | 6.9E-04 | 1.0E-01 | 3.1E-01 | 0.0E+00 | 0.0E+00 |
| 40 | 1.2E-04 | 3.5E-04 | 5.0E-04 | 1.5E-03 | 9.5E-02 | 2.8E-01 | 7.2E-05 | 2.1E-04 |
| 41 | 3.6E-04 | 1.1E-03 | 2.1E-04 | 6.3E-04 | 1.2E-03 | 3.5E-03 | 4.3E-05 | 1.3E-04 |
| 42 | 3.6E-04 | 1.1E-03 | 1.4E-04 | 4.2E-04 | 3.0E-03 | 8.8E-03 | 1.3E-03 | 3.7E-03 |
| 43 | 3.3E-04 | 9.8E-04 | 3.2E-04 | 9.4E-04 | 1.9E-03 | 5.7E-03 | 1.9E-05 | 5.5E-05 |
| 44 | 1.9E-03 | 5.6E-03 | 1.0E-06 | 3.0E-06 | 6.3E-02 | 1.9E-01 | 7.2E-05 | 2.1E-04 |
| 45 | 8.3E-04 | 2.5E-03 | 2.0E-07 | 6.0E-07 | 1.0E-01 | 3.0E-01 | 7.2E-05 | 2.1E-04 |
| 46 | 1.2E-04 | 3.5E-04 | 3.1E-06 | 9.0E-06 | 8.9E-02 | 2.6E-01 | 8.6E-05 | 2.5E-04 |
| 47 | 1.2E-03 | 3.5E-03 | 1.0E-06 | 3.0E-06 | 1.9E-01 | 5.6E-01 | 5.7E-05 | 1.7E-04 |
| 48 | 6.0E-04 | 1.8E-03 | 5.1E-06 | 1.5E-05 | 1.6E-01 | 4.8E-01 | 1.1E-04 | 3.4E-04 |
| 49 | 1.2E-04 | 3.5E-04 | 2.0E-06 | 6.0E-06 | 2.5E-01 | 7.5E-01 | 8.6E-05 | 2.5E-04 |
| 50 | 2.4E-04 | 7.0E-04 | 1.0E-06 | 3.0E-06 | 1.4E-01 | 4.2E-01 | 5.7E-05 | 1.7E-04 |
| 51 | 3.6E-04 | 1.1E-03 | 3.1E-06 | 9.0E-06 | 9.0E-02 | 2.7E-01 | 7.2E-05 | 2.1E-04 |
| 52 | 7.2E-04 | 2.1E-03 | 1.0E-06 | 3.0E-06 | 1.2E-01 | 3.5E-01 | 2.9E-05 | 8.4E-05 |
| 53 | 1.2E-03 | 3.5E-03 | 5.1E-06 | 1.5E-05 | 1.6E-01 | 4.8E-01 | 8.6E-05 | 2.5E-04 |
| 54 | 2.4E-04 | 7.0E-04 | 6.1E-06 | 1.8E-05 | 8.6E-02 | 2.5E-01 | 7.2E-05 | 2.1E-04 |
| 55 | 1.5E-03 | 4.6E-03 | 1.0E-06 | 3.0E-06 | 2.4E-02 | 7.1E-02 | 7.2E-05 | 2.1E-04 |
| 56 | 1.1E-03 | 3.2E-03 | 2.0E-06 | 6.0E-06 | 4.8E-02 | 1.4E-01 | 4.3E-05 | 1.3E-04 |
| 57 | 1.2E-04 | 3.5E-04 | 3.1E-06 | 9.0E-06 | 5.3E-02 | 1.6E-01 | 1.1E-05 | 3.4E-05 |
| 58 | 4.8E-04 | 1.4E-03 | 2.0E-06 | 6.0E-06 | 1.6E-01 | 4.7E-01 | 8.6E-05 | 2.5E-04 |
| 59 | 6.0E-04 | 1.8E-03 | 4.1E-06 | 1.2E-05 | 6.9E-03 | 2.0E-02 | 1.3E-04 | 3.8E-04 |
| 60 | 3.6E-04 | 1.1E-03 | 4.1E-06 | 1.2E-05 | 8.0E-02 | 2.4E-01 | 1.0E-04 | 3.0E-04 |
| 61 | 1.2E-03 | 3.5E-03 | 2.0E-06 | 6.0E-06 | 4.7E-02 | 1.4E-01 | 1.1E-04 | 3.4E-04 |
| 62 | 3.6E-04 | 1.1E-03 | 3.1E-06 | 9.0E-06 | 2.2E-01 | 6.5E-01 | 1.6E-04 | 4.6E-04 |
| 63 | 2.4E-05 | 7.0E-05 | 1.0E-06 | 3.0E-06 | 9.8E-02 | 2.9E-01 | 1.1E-04 | 3.4E-04 |
| 64 | 6.0E-04 | 1.8E-03 | 1.0E-06 | 3.0E-06 | 1.3E-02 | 3.8E-02 | 1.3E-04 | 3.8E-04 |
| 65 | 1.2E-03 | 3.5E-03 | 1.0E-06 | 3.0E-06 | 6.1E-02 | 1.8E-01 | 1.1E-04 | 3.4E-04 |
| 66 | 4.2E-04 | 1.2E-03 | 1.0E-06 | 3.0E-06 | 4.3E-02 | 1.3E-01 | 2.4E-04 | 7.1E-04 |
| 67 | 1.1E-04 | 3.2E-04 | 1.0E-06 | 3.0E-06 | 9.5E-02 | 2.8E-01 | 1.4E-05 | 4.2E-05 |
| 68 | 1.2E-04 | 3.5E-04 | 1.0E-06 | 3.0E-06 | 7.0E-03 | 2.1E-02 | 1.1E-05 | 3.4E-05 |
| 69 | 4.8E-04 | 1.4E-03 | 1.0E-06 | 3.0E-06 | 2.1E-02 | 6.1E-02 | 8.6E-05 | 2.5E-04 |
| **Min** | 2.4E-05 | 7.0E-05 | 2.0E-07 | 6.0E-07 | 3.0E-04 | 8.8E-04 | 0.00E+00 | 0.00E+00 |
| **Max** | 1.9E-03 | 5.6E-03 | 6.3E-04 | 1.9E-03 | 2.5E-01 | 7.5E-01 | 1.3E-03 | 3.7E-03 |
| **Average** | 5.8E-04 | 1.7E-03 | 1.5E-04 | 4.6E-04 | 6.3E-02 | 1.9E-01 | 1.1E-04 | 3.4E-04 |

Table S8. The HQ oral values in both child and adult

| Samples | **HQ_Cu_**  **(oral&adult)** | **HQ_Cu_**  **(oral&child)** | **HQ_Fe_**  **(oral&adult)** | **HQ_Fe_**  **(oral&child)** | **HQ_Mn_**  **(oral&adult)** | **HQ_Mn_**  **(oral&child)** | **HQ_Zn_**  **(oral&adult)** | **HQ_Zn_**  **(oral&child)** |
| --- | --- | --- | --- | --- | --- | --- | --- | --- |
| 1 | 1.2E-01 | 4.6E-01 | 5.6E-03 | 2.1E-02 | 6.3E-03 | 2.4E-02 | 5.0E-03 | 1.9E-02 |
| 2 | 5.3E-02 | 2.0E-01 | 8.2E-03 | 3.1E-02 | 2.5E-02 | 9.6E-02 | 5.0E-03 | 1.9E-02 |
| 3 | 7.5E-03 | 2.9E-02 | 9.0E-03 | 3.5E-02 | 5.0E-02 | 1.9E-01 | 6.0E-03 | 2.3E-02 |
| 4 | 7.5E-02 | 2.9E-01 | 3.0E-03 | 1.2E-02 | 6.4E-02 | 2.4E-01 | 4.0E-03 | 1.5E-02 |
| 5 | 3.8E-02 | 1.4E-01 | 7.7E-03 | 3.0E-02 | 6.3E-03 | 2.4E-02 | 8.0E-03 | 3.1E-02 |
| 6 | 7.5E-03 | 2.9E-02 | 9.0E-03 | 3.5E-02 | 5.0E-02 | 1.9E-01 | 6.0E-03 | 2.3E-02 |
| 7 | 1.5E-02 | 5.8E-02 | 9.9E-03 | 3.8E-02 | 2.5E-02 | 9.6E-02 | 4.0E-03 | 1.5E-02 |
| 8 | 2.3E-02 | 8.6E-02 | 6.5E-03 | 2.5E-02 | 2.5E-02 | 9.6E-02 | 5.0E-03 | 1.9E-02 |
| 9 | 4.5E-02 | 1.7E-01 | 3.9E-03 | 1.5E-02 | 2.5E-03 | 9.6E-03 | 2.0E-03 | 7.7E-03 |
| 10 | 7.5E-02 | 2.9E-01 | 2.2E-02 | 8.5E-02 | 6.3E-02 | 2.4E-01 | 6.0E-03 | 2.3E-02 |
| 11 | 1.5E-02 | 5.8E-02 | 7.7E-03 | 3.0E-02 | 1.0E-02 | 3.8E-02 | 5.0E-03 | 1.9E-02 |
| 12 | 9.8E-02 | 3.7E-01 | 5.6E-03 | 2.1E-02 | 5.0E-03 | 1.9E-02 | 5.0E-03 | 1.9E-02 |
| 13 | 6.8E-02 | 2.6E-01 | 7.3E-03 | 2.8E-02 | 1.0E-02 | 3.8E-02 | 3.0E-03 | 1.2E-02 |
| 14 | 7.5E-03 | 2.9E-02 | 4.7E-03 | 1.8E-02 | 2.5E-02 | 9.6E-02 | 8.0E-04 | 3.1E-03 |
| 15 | 3.0E-02 | 1.2E-01 | 1.3E-02 | 5.1E-02 | 4.4E-01 | 1.7E+00 | 6.0E-03 | 2.3E-02 |
| 16 | 3.8E-02 | 1.4E-01 | 1.6E-02 | 6.1E-02 | 1.2E+00 | 4.6E+00 | 9.0E-03 | 3.5E-02 |
| 17 | 2.3E-02 | 8.6E-02 | 1.4E-02 | 5.4E-02 | 1.1E+00 | 4.2E+00 | 7.0E-03 | 2.7E-02 |
| 18 | 7.5E-02 | 2.9E-01 | 2.7E-02 | 1.0E-01 | 1.2E+00 | 4.5E+00 | 8.0E-03 | 3.1E-02 |
| 19 | 2.3E-02 | 8.6E-02 | 1.3E-02 | 4.9E-02 | 5.8E-01 | 2.2E+00 | 1.1E-02 | 4.2E-02 |
| 20 | 1.5E-03 | 5.8E-03 | 5.2E-03 | 2.0E-02 | 9.8E-01 | 3.7E+00 | 8.0E-03 | 3.1E-02 |
| 21 | 3.8E-02 | 1.4E-01 | 1.6E-02 | 6.1E-02 | 1.2E+00 | 4.6E+00 | 9.0E-03 | 3.5E-02 |
| 22 | 7.5E-02 | 2.9E-01 | 2.7E-02 | 1.0E-01 | 1.2E+00 | 4.5E+00 | 8.0E-03 | 3.1E-02 |
| 23 | 2.6E-02 | 1.0E-01 | 2.0E-02 | 7.7E-02 | 8.5E-02 | 3.3E-01 | 1.7E-02 | 6.4E-02 |
| 24 | 6.8E-03 | 2.6E-02 | 1.3E-04 | 4.9E-04 | 6.3E-03 | 2.4E-02 | 1.0E-03 | 3.8E-03 |
| 25 | 5.0E-02 | 1.9E-01 | 1.1E-02 | 4.4E-02 | 5.9E-01 | 2.3E+00 | 1.6E-03 | 6.1E-03 |
| 26 | 4.1E-02 | 1.6E-01 | 9.3E-03 | 3.5E-02 | 9.7E-02 | 3.7E-01 | 2.6E-03 | 1.0E-02 |
| 27 | 3.8E-02 | 1.5E-01 | 1.1E-02 | 4.1E-02 | 3.1E-02 | 1.2E-01 | 9.0E-04 | 3.5E-03 |
| 28 | 4.0E-02 | 1.5E-01 | 1.3E-02 | 5.1E-02 | 6.5E-02 | 2.5E-01 | 4.3E-02 | 1.6E-01 |
| 29 | 3.9E-02 | 1.5E-01 | 7.8E-03 | 3.0E-02 | 6.6E-01 | 2.5E+00 | 6.0E-04 | 2.3E-03 |
| 30 | 9.0E-03 | 3.5E-02 | 3.7E-03 | 1.4E-02 | 1.0E+00 | 3.9E+00 | 5.0E-04 | 1.9E-03 |
| 31 | 3.8E-02 | 1.5E-01 | 9.8E-03 | 3.7E-02 | 1.2E+00 | 4.4E+00 | 8.0E-04 | 3.1E-03 |
| 32 | 4.1E-02 | 1.6E-01 | 7.0E-03 | 2.7E-02 | 1.1E-01 | 4.2E-01 | 3.6E-03 | 1.4E-02 |
| 33 | 3.8E-03 | 1.4E-02 | 2.6E-04 | 9.9E-04 | 1.3E-02 | 4.8E-02 | 8.0E-04 | 3.1E-03 |
| 34 | 2.6E-02 | 1.0E-01 | 8.7E-03 | 3.3E-02 | 1.9E-02 | 7.2E-02 | 1.3E-03 | 5.0E-03 |
| 35 | 3.6E-02 | 1.4E-01 | 8.5E-03 | 3.2E-02 | 1.1E-01 | 4.2E-01 | 3.6E-03 | 1.4E-02 |
| 36 | 1.5E-02 | 5.8E-02 | 1.1E-02 | 4.1E-02 | 5.7E-01 | 2.2E+00 | 1.2E-02 | 4.6E-02 |
| 37 | 3.8E-03 | 1.4E-02 | 3.0E-04 | 1.2E-03 | 7.5E-03 | 2.9E-02 | 3.0E-04 | 1.2E-03 |
| 38 | 4.5E-03 | 1.7E-02 | 1.1E-02 | 4.3E-02 | 5.1E-01 | 2.0E+00 | 7.0E-03 | 2.7E-02 |
| 39 | 1.5E-02 | 5.8E-02 | 9.9E-03 | 3.8E-02 | 8.8E-01 | 3.4E+00 | 0.0E+00 | 0.0E+00 |
| 40 | 7.5E-03 | 2.9E-02 | 2.1E-02 | 8.1E-02 | 8.0E-01 | 3.1E+00 | 5.0E-03 | 1.9E-02 |
| 41 | 2.3E-02 | 8.6E-02 | 9.0E-03 | 3.5E-02 | 1.0E-02 | 3.8E-02 | 3.0E-03 | 1.2E-02 |
| 42 | 2.3E-02 | 8.6E-02 | 6.0E-03 | 2.3E-02 | 2.5E-02 | 9.6E-02 | 8.8E-02 | 3.4E-01 |
| 43 | 2.1E-02 | 8.1E-02 | 1.3E-02 | 5.1E-02 | 1.6E-02 | 6.2E-02 | 1.3E-03 | 5.0E-03 |
| 44 | 1.2E-01 | 4.6E-01 | 4.3E-05 | 1.6E-04 | 5.3E-01 | 2.0E+00 | 5.0E-03 | 1.9E-02 |
| 45 | 5.3E-02 | 2.0E-01 | 8.6E-06 | 3.3E-05 | 8.5E-01 | 3.2E+00 | 5.0E-03 | 1.9E-02 |
| 46 | 7.5E-03 | 2.9E-02 | 1.3E-04 | 4.9E-04 | 7.5E-01 | 2.9E+00 | 6.0E-03 | 2.3E-02 |
| 47 | 7.5E-02 | 2.9E-01 | 4.3E-05 | 1.6E-04 | 1.6E+00 | 6.1E+00 | 4.0E-03 | 1.5E-02 |
| 48 | 3.8E-02 | 1.4E-01 | 2.2E-04 | 8.2E-04 | 1.4E+00 | 5.2E+00 | 8.0E-03 | 3.1E-02 |
| 49 | 7.5E-03 | 2.9E-02 | 8.6E-05 | 3.3E-04 | 2.1E+00 | 8.2E+00 | 6.0E-03 | 2.3E-02 |
| 50 | 1.5E-02 | 5.8E-02 | 4.3E-05 | 1.6E-04 | 1.2E+00 | 4.6E+00 | 4.0E-03 | 1.5E-02 |
| 51 | 2.3E-02 | 8.6E-02 | 1.3E-04 | 4.9E-04 | 7.6E-01 | 2.9E+00 | 5.0E-03 | 1.9E-02 |
| 52 | 4.5E-02 | 1.7E-01 | 4.3E-05 | 1.6E-04 | 1.0E+00 | 3.9E+00 | 2.0E-03 | 7.7E-03 |
| 53 | 7.5E-02 | 2.9E-01 | 2.2E-04 | 8.2E-04 | 1.4E+00 | 5.2E+00 | 6.0E-03 | 2.3E-02 |
| 54 | 1.5E-02 | 5.8E-02 | 2.6E-04 | 9.9E-04 | 7.2E-01 | 2.8E+00 | 5.0E-03 | 1.9E-02 |
| 55 | 9.8E-02 | 3.7E-01 | 4.3E-05 | 1.6E-04 | 2.0E-01 | 7.8E-01 | 5.0E-03 | 1.9E-02 |
| 56 | 6.8E-02 | 2.6E-01 | 8.6E-05 | 3.3E-04 | 4.0E-01 | 1.5E+00 | 3.0E-03 | 1.2E-02 |
| 57 | 7.5E-03 | 2.9E-02 | 1.3E-04 | 4.9E-04 | 4.4E-01 | 1.7E+00 | 8.0E-04 | 3.1E-03 |
| 58 | 3.0E-02 | 1.2E-01 | 8.6E-05 | 3.3E-04 | 1.3E+00 | 5.1E+00 | 6.0E-03 | 2.3E-02 |
| 59 | 3.8E-02 | 1.4E-01 | 1.7E-04 | 6.6E-04 | 5.8E-02 | 2.2E-01 | 9.0E-03 | 3.5E-02 |
| 60 | 2.3E-02 | 8.6E-02 | 1.7E-04 | 6.6E-04 | 6.7E-01 | 2.6E+00 | 7.0E-03 | 2.7E-02 |
| 61 | 7.5E-02 | 2.9E-01 | 8.6E-05 | 3.3E-04 | 3.9E-01 | 1.5E+00 | 8.0E-03 | 3.1E-02 |
| 62 | 2.3E-02 | 8.6E-02 | 1.3E-04 | 4.9E-04 | 1.9E+00 | 7.1E+00 | 1.1E-02 | 4.2E-02 |
| 63 | 1.5E-03 | 5.8E-03 | 4.3E-05 | 1.6E-04 | 8.3E-01 | 3.2E+00 | 8.0E-03 | 3.1E-02 |
| 64 | 3.8E-02 | 1.4E-01 | 4.3E-05 | 1.6E-04 | 1.1E-01 | 4.1E-01 | 9.0E-03 | 3.5E-02 |
| 65 | 7.5E-02 | 2.9E-01 | 4.3E-05 | 1.6E-04 | 5.1E-01 | 2.0E+00 | 8.0E-03 | 3.1E-02 |
| 66 | 2.6E-02 | 1.0E-01 | 4.3E-05 | 1.6E-04 | 3.6E-01 | 1.4E+00 | 1.7E-02 | 6.4E-02 |
| 67 | 6.8E-03 | 2.6E-02 | 4.3E-05 | 1.6E-04 | 8.0E-01 | 3.0E+00 | 1.0E-03 | 3.8E-03 |
| 68 | 7.5E-03 | 2.9E-02 | 4.3E-05 | 1.6E-04 | 5.9E-02 | 2.3E-01 | 8.0E-04 | 3.1E-03 |
| 69 | 3.0E-02 | 1.2E-01 | 4.3E-05 | 1.6E-04 | 1.7E-01 | 6.6E-01 | 6.0E-03 | 2.3E-02 |
| **Min** | 1.5E-03 | 5.8E-03 | 8.6E-06 | 3.3E-05 | 2.5E-03 | 9.6E-03 | 0.0E+00 | 0.0E+00 |
| **Max** | 1.2E-01 | 4.6E-01 | 2.7E-02 | 1.0E-01 | 2.1E+00 | 8.2E+00 | 8.8E-02 | 3.4E-01 |
| **Average** | 3.7E-02 | 1.4E-01 | 6.5E-03 | 2.5E-02 | 5.3E-01 | 2.0E+00 | 8.0E-03 | 3.1E-02 |
